# Piperine–Chlorogenic Acid Hybrid Inhibits the Proliferation of the SK-MEL-147 Melanoma Cells by Modulating Mitotic Kinases

**DOI:** 10.3390/ph16020145

**Published:** 2023-01-19

**Authors:** Carolina Girotto Pressete, Flávia Pereira Dias Viegas, Thâmara Gaspar Campos, Ester Siqueira Caixeta, João Adolfo Costa Hanemann, Guilherme Álvaro Ferreira-Silva, Bruno Zavan, Alexandre Ferro Aissa, Marta Miyazawa, Claudio Viegas-Jr, Marisa Ionta

**Affiliations:** 1 Institute of Biomedical Sciences, Federal University of Alfenas, Alfenas 37130-001, MG, Brazil; 2 Institute of Chemistry, Laboratory of Research in Medicinal Chemistry, Federal University of Alfenas, Alfenas 37133-840, MG, Brazil; 3 Department of Clinic and Surgery, School of Dentistry, Federal University of Alfenas, Alfenas 37130-001, MG, Brazil

**Keywords:** antiproliferative activity, cell cycle arrest, apoptosis, CDKN1A, CCNB1, aurora kinases

## Abstract

Melanoma is considered the most aggressive form of skin cancer, showing high metastatic potential and persistent high mortality rates despite the introduction of immunotherapy and targeted therapies. Thus, it is important to identify new drug candidates for melanoma. The design of hybrid molecules, with different pharmacophore fragments combined in the same scaffold, is an interesting strategy for obtaining new multi-target and more effective anticancer drugs. We designed nine hybrid compounds bearing piperine and chlorogenic acid pharmacophoric groups and evaluated their antitumoral potential on melanoma cells with distinct mutational profiles SK-MEL-147, CHL-1 and WM1366. We identified the compound named PQM-277 (**3a**) to be the most cytotoxic one, inhibiting mitosis progression and promoting an accumulation of cells in pro-metaphase and metaphase by altering the expression of genes that govern G2/M transition and mitosis onset. Compound **3a** downregulated *FOXM1*, *CCNB1*, *CDK1*, *AURKA*, *AURKB*, and *PLK1*, and upregulated *CDKN1A*. Molecular docking showed that **3a** could interact with the CUL1-RBX1 complex, which activity is necessary to trigger molecular events essential for FOXM1 transactivation and, in turn, G2/M gene expression. In addition, compound **3a** effectively induced apoptosis by increasing *BAX/BCL2* ratio. Our findings demonstrate that **3a** is an important antitumor candidate prototype and support further investigations to evaluate its potential for melanoma treatment, especially for refractory cases to BRAF/MEK inhibitors.

## 1. Introduction

Melanoma is an aggressive malignant tumor responsible for 75% of the deaths caused by skin cancer. The lethality of cutaneous malignant melanoma is associated with their high capacity to form metastases [1,2]. The intrinsic or acquired resistance of tumor cells to currently available drugs represents an important obstacle to overcome. In general, melanomas have more mutations than any other cancer type, and *BRAF, NRAS, CDKN2A* and *TP53* mutations are frequently observed in cutaneous melanoma [3,4].

Therapeutic approaches for melanoma include surgical removal, radiotherapy, chemotherapy, targeted therapy, and immunotherapy. Dacarbazine has been used in traditional chemotherapy to treat melanoma patients since 1975, with few long-lasting responses (lower than 2%) [5,6]. Temozolomide, an analogue of dacarbazine, has been proposed for the treatment of melanoma brain metastases, but with few improvements in overall survival [7]. Currently, these cytotoxic drugs are used as a second-line treatment in combination with other drugs. Importantly, the understanding of melanoma biology at the cellular and molecular levels has allowed the identification of therapeutic targets, prompting the introduction of kinase and immune checkpoint inhibitors into therapeutic protocols, substantially improving the 5-year survival rate [8,9].

Hyperactivation of the MAPK (mitogen-activated protein kinase) RAF/MEK/ERK signaling pathway is commonly observed in cutaneous melanomas due to *NRAS* or *BRAF* mutations [3,4], and systemic treatment in these cases may include BRAF and MEK inhibitors. Several studies have shown that the combination of BRAF and MEK inhibitors is more effective compared to the isolated use of BRAF inhibitors [10,11]. There is evidence that a combined-drug regimen improves the therapeutic response and may contribute to overcoming tumor resistance. In this way, the design of hybrid molecules has emerged as an interesting strategy for obtaining new multi-target and potentially more effective anticancer drugs [12].

**Figure 1:**
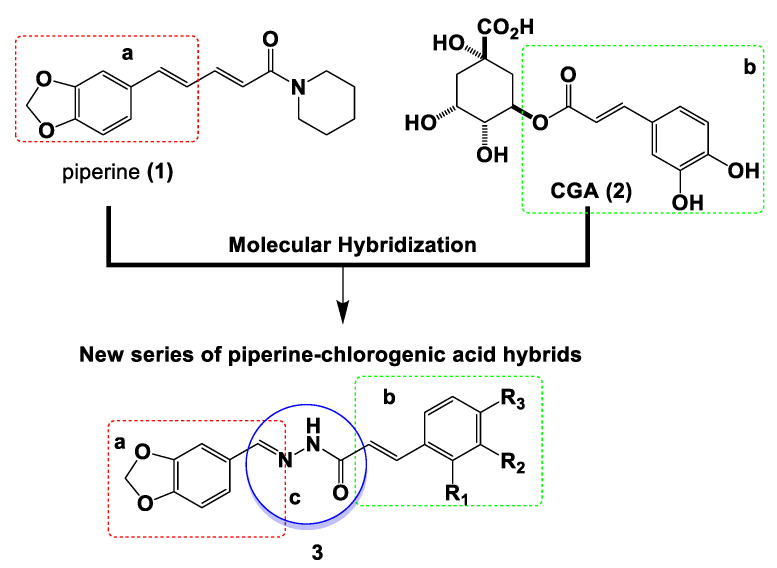
Design of new piperine–chlorogenic acid acylhydrazone hybrid series **3** by molecular hybridization of structural fragments from piperine (**1**) and chlorogenic acid (**2**, CGA) connected by an acylhydrazone subunit. conjugated benzo[d]1,3-dioxole moiety from the piperine (**1**); cinnamoyl fragment from chlorogenic acid (**2**); acylhydrazone spacer subunit. ^a^ conjugated benzo[d]1,3-dioxole moiety from the piperine (**1**); ^b^ cinnamoyl fragment from chlorogenic acid (**2**); ^c^ acylhydrazone spacer subunit.

Natural products with pharmacological potential have been used as prototypes for the design of new drugs, including anticancer candidates [13]. Piperine (**1**, Figure 1) is an alkaloid found in many plant species, and is especially abundant in the *Piper* genus (e.g., *Piper nigrum*), with several biological properties, such as antioxidant, anti-inflammatory, anti-angiogenic, anti-parasitic, and antitumor activities [14,15,16]. Regarding the antitumor activity of piperine, different mechanisms of action have been proposed, including oxidative stress, cell cycle arrest, and apoptosis induction [17]. In silico studies have predicted that piperine could interact with cell-cycle regulators (cyclin A or CDK2) and proteins that govern apoptosis process (BCL-xL) [18]. Likewise, chlorogenic acid (CGA, **2**, Figure 1) is a natural phenolic compound, also abundant in many plant species [19], that has been extensively reported to possess antioxidant, anti-inflammatory, and antitumor properties [19,20,21]. Similar to piperine, CGA induces cell cycle arrest and apoptosis in tumor cell lines by acting on different molecular targets [22,23].

Despite previous data regarding biological activity of the piperine and CGA, there are few systematic structure–activity relationship studies evidencing their pharmacophoric contributions for anticancer activity. This information is critical for addressing the rational design of novel drug candidates with improved druggability [24]. The structure of **1** can be categorized into three regions: (i) an aromatic ring moiety; (ii) an aliphatic unsaturated chain; and (iii) the piperidine ring. All these structural subunits have been well explored by medicinal chemists aiming to obtain innovative compound with improved antitumor activity [25]. Regarding CGA, to the best of our knowledge, there have been no structure–activity studies. In general, the biological activities of this compound and its derivatives have been attributed mainly to the presence of the catechol-like and polyphenol-based caffeoyl moiety [19].

Considering the antitumor potential of the natural compounds **1** and **2**, and our continuous efforts to search new small molecules as potential drug candidates for the treatment of malignant tumors, a new series of piperine–CGA hybrids was designed, and their antiproliferative activity was evaluated on melanoma cell lines with distinct mutation profiles. The rationale of the molecular hybridization-based strategy in the conception of the piperine–CGA hybrid scaffold took into account the preservation of the conjugated benzo[d]1,3-dioxole moiety (**a**, Figure 1)) from the parent piperine structure, connected to a substituted cinnamoyl fragment (**b**, Figure 1) by an acylhydrazone spacer subunit (**c**, Figure 1), generating a piperonyl–cynnamoyl–acylhydrazone feature, varying the nature of the substituents on the aromatic ring conjugated to the α,β-unsaturated ketone fragment. In this series, most compounds have at least one Oxygen substituent (hydroxyl or methoxyl) at different positions in the aromatic ring (**b**, Figure 1), as well as the unsubstituted ring or with electronegative substituents such as chlorine and trifluoromethyl group aiming to evaluate their contribution to the antiproliferative activity.

## 2. Results

### 2.1. Chemistry

**Scheme 1:**
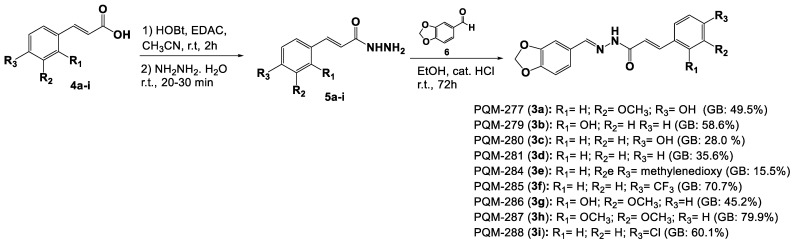
Synthetic route and global yields target-compounds **3a–i**.

The synthetic route for the target compounds **3a–i** is detailed in Scheme 1. The synthesis was performed by means of a two-step synthetic approach, using a series of substituted cinnamoyl acids (**4a–i**) as starting materials, which were reacted with hydrazine monohydrate in the presence of hydroxybenzotriazole (HOBt) and 1-ethyl-3-(3-dimethylaminopropyl) carbodiimide (EDAC) used as coupling reagent mixture in acetonitrile, generating an activated benzotriazole ester adduct in situ that reacts with hydrazine by an acyl substitution reaction, leading to the key-hydrazide intermediates **5a–i** in 28–87% yields (Scheme 1). In a second step, hydrazides **5a–i** were then coupled with piperonal (**6**) in a solution of ethanol and using conc. HCl as a catalyst, furnishing the desired series of piperonyl-cinnamoyl acylhydrazones **3a–i** in 35–91% yields, after purification by recrystallization or column chromatography. All spectra are available in the Supplementary Materials.

### 2.2. Compound PQM-277 (***3a***) Is Cytotoxic to Melanoma Cells but Not to Primary Dermal Fibroblast

**Figure 2:**
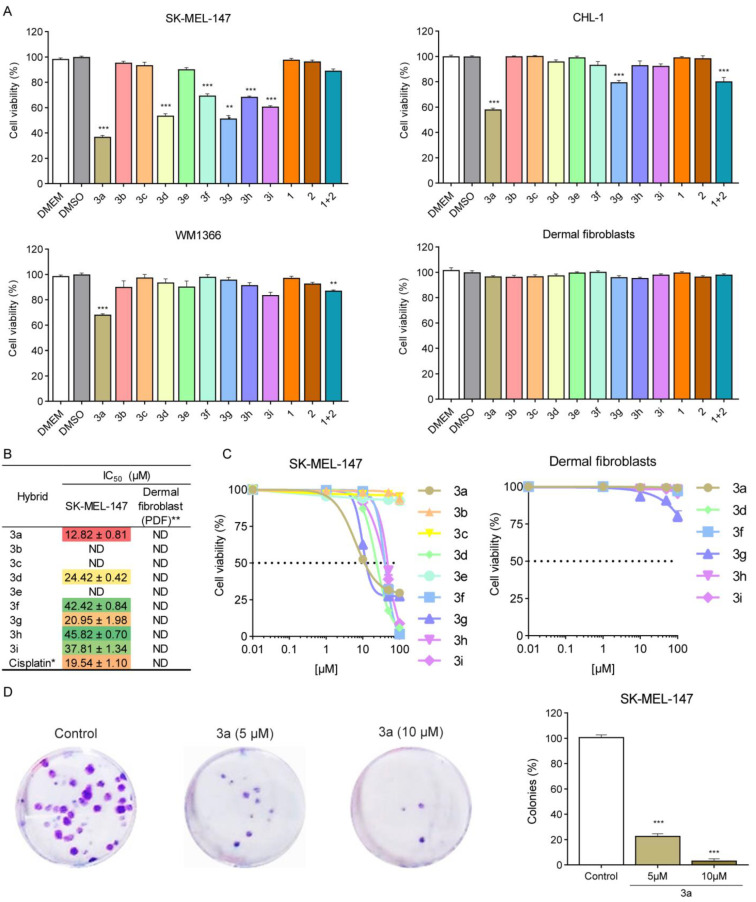
Compound **3a** is cytotoxic to melanoma cells but not to primary dermal fibroblast. (**A**) Cell viability was determined by sulforhodamine B (SRB) after 48 h of treatment with the different compounds. DMSO (0.1% *v*/*v*) was used as negative control; **1**, piperine; **2**, chlorogenic acid. (**B**) IC_50_ values (µM) determined by SRB. Cell cultures were treated with substances **3a–i** for 48 h. * Cisplatin was used as a positive control. ** non-tumor cell line (primary dermal fibroblast). ND: not determined once cell viability was not sufficiently reduced to determine IC_50_ values. (**C**) Growth curves determined by SRB after 48 h of treatment with target-compounds in SK-MEL-147 cells and dermal fibroblast cell cultures. (**D**) Clonogenic assay of SK-MEL-147 cells treated with **3a** at 5 or 10 µM for 24 h and recovered in fresh medium for additional 12 days. ** *p* < 0.01 and *** *p* < 0.001 compared to DMSO according to ANOVA followed by Dunnet post-test. DMSO: Dimethylsulfoxide; DMEM: Dulbecco’s Modified Eagle Medium.

We evaluated the cytotoxic activity of the nine synthetic piperine–chlorogenic acid acylhydrazone hybrid derivatives (**3a–i**) against three melanoma cell lines (Figure 2). Cell cultures were treated with different compounds for 48 h and relative viability rate was determined by sulforhodamine B (SRB) assay (Figure 2A). SK-MEL-147 cell line was more responsive to the tested compounds than CHL-1 or WM1366 cell lines. Reduced cell viability was observed in SK-MEL-147 cells treated with PQM-277 (**3a**), PQM-281 (**3d**), PQM-285 (**3f**), PQM-286 (**3g**), PQM-287 (**3h**) and PQM-288 (**3i**) (Figure 2A). However, PQM-227 (**3a**), PQM-286 (**3g**), and PQM-281 (**3d**) were identified as the most active compounds. We did not observe a reduction in cell viability in SK-MEL-147 cells treated with the parent compounds piperine or chlorogenic acid in the same experimental conditions. Likewise, cell viability was not altered in cells treated with a physical mixture of piperine and chlorogenic acid. Interestingly, the compounds had minimal effect on primary dermal fibroblast, indicating no cytotoxic effect of these compounds on normal cells. Based on these preliminary data, SK-MEL-147 was selected for further investigations. The IC_50_ values were determined for all nine tested compounds at 48 h post treatment. We confirmed that PQM-277 (**3a**) was the most potent cytotoxic compound against SK-MEL-147 with IC_50_ = 12.82 µM, even lower than cisplatin (IC_50_ = 19.54 µM), a powerful cytotoxic antineoplastic agent (Figure 2B,C), followed by derivatives PQM-286 (**3g**) and PQM-281 (**3d**) with IC_50_ values of 20.95 and 24.42 µM, respectively. The IC_50_ values of the tested compounds were not determined on fibroblasts, since cell viability was minimally affected by the treatment (Figure 2C). Regarding compound **3d**, it has been already reported by Carvalho et al. [26] as a part of a project seeking for novel antitrypanosomal ligands. The capacity of **3a** to inhibit proliferation of SK-MEL-147 cells was initially investigated through clonogenic assay. We demonstrated that **3a** has antiproliferative activity on SK-MEL-147 once the frequency of colonies was significantly reduced in treated samples when compared to control groups (Figure 2D). Thus, additional experimental approaches were performed to investigate deeply the effects of the **3a** on proliferative behavior of SK-MEL-147 cells.

### 2.3. Compound PQM-277 (***3a***) Induces Mitotic Arrest at Prometaphase in SK-MEL-147 Cells

**Figure 3:**
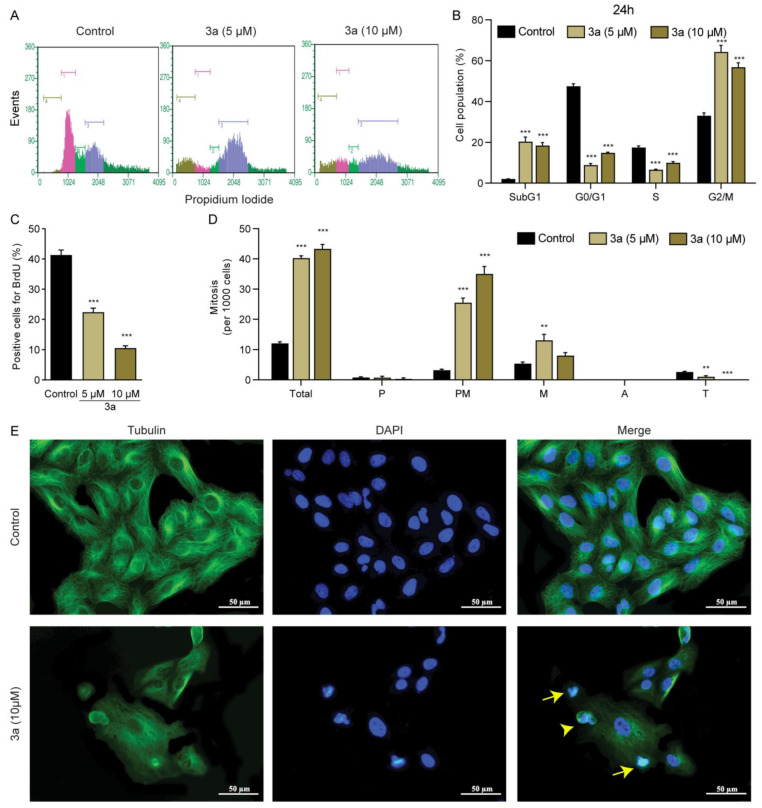
Compound **3a** induced mitotic arrest at prometaphase in SK-MEL-147 cells. (**A**,**B**) Representative histograms obtained by flow cytometry and cell cycle analysis. SK-MEL-147 cultures were treated for 24 h with **3a** at 5 and 10 µM. Sub-G1 (brown), G0/G1 (pink), S (green), G2/M (blue), hypertetraploid population (dark green). (**C**) BrdU-positive cells determined after 24 h of treatment. (**D**) Mitotic indices determined after 24 h of treatment by counting the mitosis from cytological preparations labeled with DAPI and anti-α-tubulin. ** *p* < 0.01 and *** *p <* 0.001 according to ANOVA followed by Dunnet post-test compared with the control group. (**E**) Representative images of cells treated with **3a** at 10 µM showing abnormal spindle formation (arrowhead) and metaphase with failure of chromosome alignment at metaphase (arrows). DAPI: 4′,6-diamidino-2-phenylindole.

Cell cycle analysis was performed to characterize the antiproliferative activity of **3a** in SK-MEL-147 cells. We observed a cell cycle arrest at G2/M in SK-MEL-147 cells treated with 5 or 10 µM for 24 h (Figure 3A,B). There was a significant increase in G2/M population with reduction in the frequency of cells in S-phase (Figure 3A,B), which was consistent with the lower frequency of cells labelled with BrdU, a specific S-phase marker (Figure 3C). Compound **3a** also increased the frequency of cells in the Sub-G1 phase, suggesting that after 24 h of treatment, a percentage of cells could be dying through apoptosis [27]. Mitotic indices showed that **3a** increased the frequency of cells in both prometaphase and metaphase stages. Importantly, no cells were observed in anaphase and there was reduction in the frequency of cells in telophase (Figure 3D). In addition, it was possible to observe that the dynamic of polymerization of the microtubule seems to have been affected by **3a** treatment (Figure 3E) leading to failure of chromosome alignment at metaphasic plate. Therefore, the compound **3a** induced mitotic arrest possibly disrupting the regulatory mechanism associated with the events required to mitosis onset (Figure 3E, lower panel).

### 2.4. Compound PQM-277 (***3a***) Increased the Expression of p21 and Decreased the Expression of Aurora Kinases

**Figure 4:**
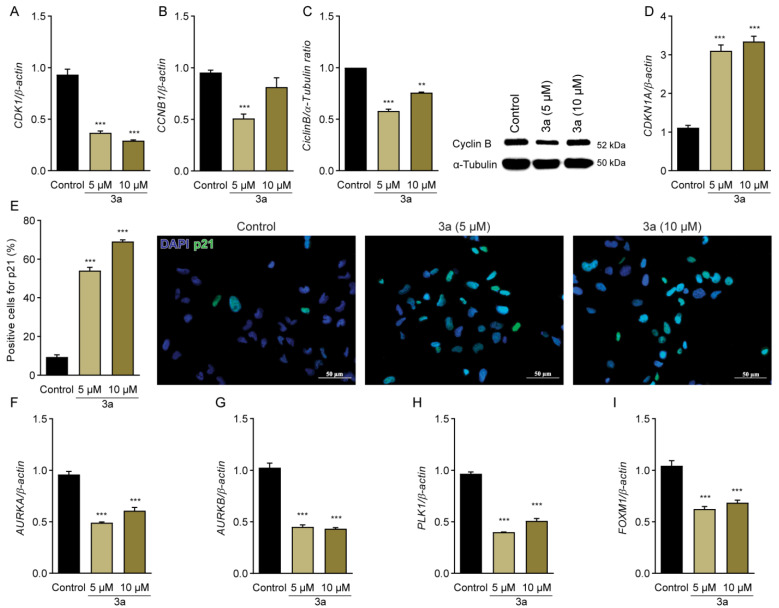
Compound **3a** increased the expression of p21 and decreased the expression of aurora kinases. SK-MEL-147 cells were treated with **3a** at 5 and 10 µM for 24 h and relative expression of target genes was evaluated by qPCR (**A**–**I**), immunoblot (**C**), or immunocytochemistry (**E**). (**A**) Relative CDK1 mRNA expression; (**B**) Relative CCNB1 mRNA expression; (**C**) Relative CCNB1 at protein levels; (**D**) Relative CDKN1A mRNA expression; (**E**) Frequency of p21-positive cells determined by immunofluorescence. Blue: DAPI; green: p21. (**F**) Relative AURKA mRNA expression; (**G**) Relative AURKB mRNA expression; (**H**) Relative PLK1 mRNA expression; (**I**) Relative FOXM1 mRNA expression. ** *p* < 0.01 and *** *p* < 0.001 according to ANOVA followed by Dunnet post-test compared with control group.

In order to investigate the potential molecular targets involved in the antiproliferative/cytotoxic activity of **3a**, we evaluated the expression of the key regulators of G2/M transition (Figure 4) [28,29]. Cyclin B-dependent kinase (CDK1)-CCNB complex is responsible for the correct transition from G2 to mitosis [30]. Gene expression analysis showed that **3a** downregulated CDK1 (Figure 4A). CCNB1 was also downregulated at the mRNA (Figure 4B) and protein levels (Figure 4C). This effect was accompanied by an increase in p21, which is responsible for inactivating the complex CDK1-CCNB (Figure 4D,E) [31].

Furthermore, we investigated gene expression profiles of AURKA, AURKB and PLK1, which are also involved in G2/M transition [32]. We found a reduced abundance of mRNA for AURKA (Figure 4F), AURKAB (Figure 4G) and PLK1 (Figure 4H) in **3a**-treated cells. In addition, there was downregulation of forkhead box protein M1 (FOXM1) (Figure 4I), a transcription factor that regulates several genes associated with cell cycle, including AURKA, AURKB and PLK1 [33]. These data reinforce that **3a** inhibits proliferation of SK-MEL-147 and this effect was associated with its ability to modulate critical regulators of cell cycle progression.

### 2.5. Compound PQM-277 (***3a***) Induced Apoptosis in SK-MEL-147 Cells

**Figure 5:**
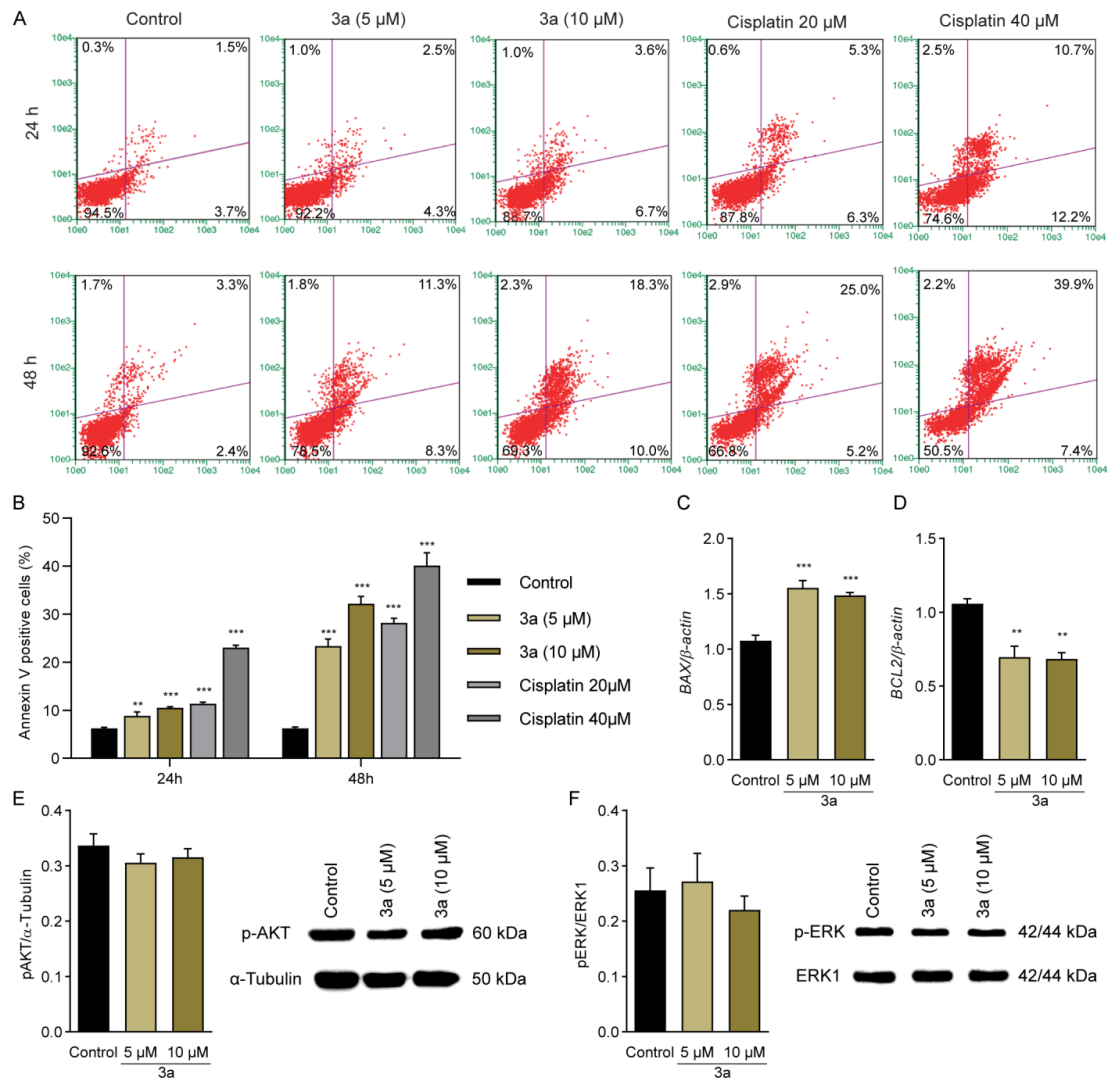
Compound **3a** induced apoptosis in SK-MEL-147 cells. (**A**,**B**) Representative dot plots and analysis of annexin V/7-AAD assay performed in SK-MEL-147 cells after 24 and 48 h of treatment with **3a**. Viable cells (lower left quadrants), early apoptosis (lower right quadrants), late apoptosis (upper right quadrants), and necrotic cells (upper left quadrants); (**C**,**D**) Relative mRNA expression of BAX, respectively, determined by qPCR in SK-MEL-147 cells treated with **3a** at 5 and 10 µM for 24 h; (**E**,**F**) Relative protein levels of p-AKT (**E**) and p-ERK (**F**) by immunoblot in SK-MEL-147 cells treated for 24 h with **3a** at 5 and 10 µM. α-tubulin and ERK-1 were used as a loading controls; ** *p* < 0.01 and *** *p* < 0.001 according to ANOVA followed by Dunnet post-test compared with control group.

Considering the significant increase in the sub-G1 population previously observed by cell cycle analysis (Figure 3A,B), we evaluated the pro-apoptotic potential of **3a** in SK-MEL-147 cells using annexin V assay (Figure 5). Cisplatin was used as a positive control to validate data obtained by flow cytometry. We found an increased frequency of annexin V-positive cells in samples treated with **3a** at 5 and 10 μM for 24 or 48 h (Figure 5A,B). Moreover, we observed an increase in the expression of the pro-apoptotic gene BAX (Figure 5C) and a decrease in the expression of the anti-apoptotic gene BCL2 (Figure 5D). It is known that cells under treatment with antiproliferative drugs could modulate ERK and AKT signaling pathway [34,35]. However, by measuring the expression levels of these proteins, no alteration in p-AKT (Figure 5E) and p-ERK (Figure 5F) was observed under **3a** treatment.

### 2.6. G2/M Arrest and Cell Death Induced by PQM-277 (***3a***) Is Not Associated with DNA Damage

**Figure 6:**
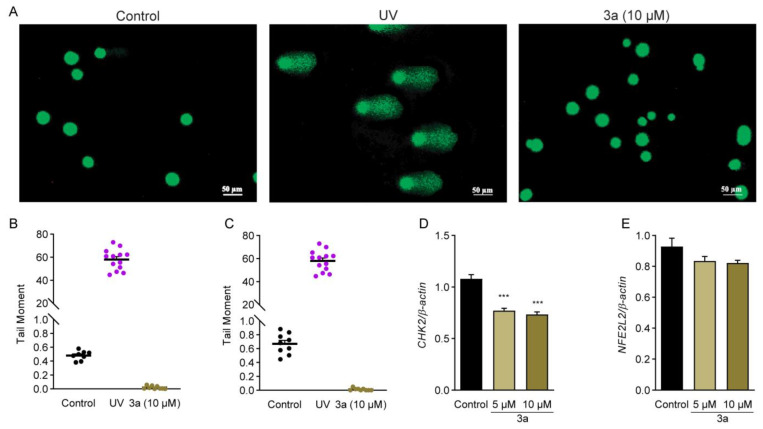
G2/M arrest and cell death induced by 3a is not associated with DNA damage. (**A**) Representative images of the nucleoids from the cells treated with **3a** for 24 h analyzed by Comet assay. (**B**,**C**) Comet assay analysis showing DNA damage indices represented by the Tail Moment after 12 and 24 h of treatment with **3a**. UV radiation was used as a positive control. (**D**,**E**) Relative mRNA expression of CHK2 and NFE2L2, respectively, determined by qPCR in SK-MEL-147 cells treated with **3a** at 5 or 10 µM for 24 h. *** *p* < 0.001 according to ANOVA followed by Dunnet post-test compared with control group.

We investigated whether pro-apoptotic activity of **3a** in SK-MEL-147 melanoma cells was triggered by DNA damage induced by the treatment. Thus, comet assay was performed to evaluate DNA damage events (Figure 6). Cells irradiated with ultraviolet (UV) were used as positive control and showed increased DNA damage (Figure 6A–C). On the other hand, the treatment with **3a** at 10 μM for 12 h (Figure 6A,B) or 24h (Figure 6C) did not induce DNA damage. G2/M arrest can be induced by the activation of CHK2 that sustain, but do not trigger, G2 arrest induced by DNA damage [36]. Moreover, CHK2 is one of the key genes responsible for the DNA damage response, and changes in its expression could indicate a DNA damage response to repair DNA. Our results showed decreased expression of CHK2 by **3a** treatment (Figure 6D), indicating that no DNA damage response was triggered under these conditions. This evidence was confirmed by the lack of changes in the expression of NFE2L2 (Figure 6E) the critical gene in response to oxidative stress [37]. Taken together, these results suggest that G2M arrest induced by **3a** in SK-MEL-147 cells is not associated with DNA damage.

### 2.7. Molecular Docking Predicts the Ligation of PQM-277 (***3a***) to a CUL1 Degradation Complex

**Figure 7:**
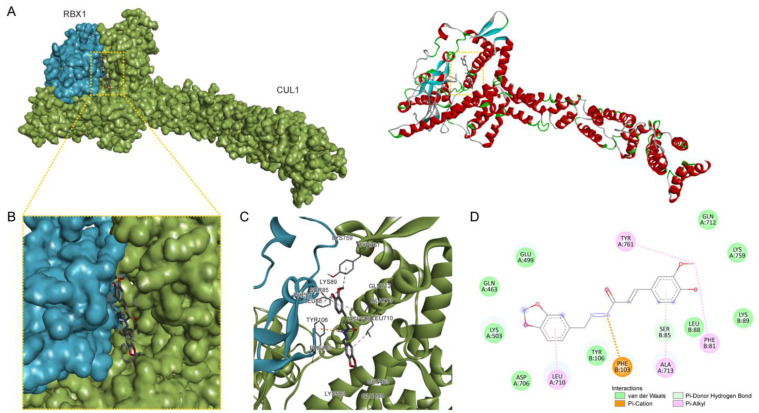
Molecular docking of **3a** into the CUL1-RBX1 complex. (**A**) Overall view of CUL1-RBX1 complex bound with **3a**, shown in the yellow dashed square inside the ligand surface (left) and inside the secondary structure (right). (**B**) Close-up view of interaction between **3a** and the CUL1-RBX1 receptor surface. (**C**,**D**) Detailed interactions of **3a** with the receptor amino acids shown in three (**C**) and two dimensions (**D**).

Since we observed that cells treated with **3a** were arrested in G2/M and showed decreased expression of late cell cycle genes, we hypothesized that **3a** could interfere with the control of the expression of these genes. Late cell cycle gene expression is controlled by the multi-vulval class B (MuvB) complex, which first interacts with BMYB (encoded by MYB-like 2, MYBL2) to express genes responsible for the initial stages of G2/M and later interacts with FOXM1 to initiate the expression of late G2/M genes [38]. The degradation of BMYB upon ligation of FOXM1 is crucial to the correct expression of late cell cycle genes controlled by FOXM1 [39]. BMYB is degraded by the cullin 1 (CUL1)-dependent E3 ligase [40]. For this reason, we predicted the ligation of **3a** to the (CUL1)-dependent E3 ligase complex using molecular docking [41] (Figure 7A–C). The predicted free energy of the receptor–ligand binding was −8.3 kcal mol–1 for the first pose, which was used for further analysis. The interactions were predicted to be with both proteins CUL1 and RBX1 of the complex (Figure 7C,D). Compound **3a** docked via Van der Waals interactions with GLN463, GLU499, LYS503, ASP706, GLN712, and LYS759, and via pi-alkyl interactions with LEU710, ALA713, TYR761 of CUL1. The interactions with RBX1 protein were predicted to be via Van der Waals interactions with LEU88, LYS89, and TYR106, via pi-cation with PHE103, via pi-donor hydrogen bond with SER85, and via pi-alkyl with PHE81.

## 3. Discussion

In the present study, we designed nine hybrid compounds that display innovative chemical structures bearing fragments of piperine and chlorogenic acid connected by an N-acylhydrazone subunit, and evaluated their antitumor potential on melanoma cell lines SK-MEL-147, CHL-1 and WM1366, which are representative of different melanoma subtypes due to their mutational profiles [42,43,44]. The lethality of the malignant melanomas was correlated with their high metastatic potential and drug resistance [45]. Thus, the development of new drugs that could suppress tumor progression and overcome drug resistance is critical for the improvement of melanoma treatment.

SK-MEL-147 was more sensitive to the treatments than WM1366 and CHL-1 cells. SK-MEL-147 cells harbor an *NRAS* mutation, which represents the second most frequent genetic mutation of cutaneous melanoma [42,46]. Mutations in *NRAS* gene have been correlated with poorer overall survival [47], and there is an unmet clinical urge for effective therapeutic approach for patients with this melanoma subtype. Among the tested compounds, derivatives **3a**, **3d**, **3f**, **3g**, **3h**, and **3i** reduced the viability of SK-MEL-147 cells, and **3a** was the most cytotoxic compound. Interestingly, the structure–activity relationship analysis among all nine piperine–chlorogenic acid acylhydrazone hybrid derivatives revealed that the presence of a methoxy-hydroxy disubstituted cinnamoyl subunit was an important feature for the antiproliferative activity. In addition, the regiochemistry of these two substituents seems to be crucial for the cytotoxic potency against SK-MEL-147 cells, with the 4-hydroxy-3-methoxy-substituted derivative **3a** being almost 2-fold more potent than its 2-hydroxy-3-methoxy regioisomer **3g**.

The cytotoxicity of **3a** on SK-MEL-147 cells was higher than cisplatin or its parent compounds piperine and chlorogenic acid. These results are in accordance with previous reports in which the cytotoxic activity of the piperine or chlorogenic acid was demonstrated only when these compounds were assayed at high concentrations [48,49]. We also observed that cell viability was minimally reduced when samples were treated with a combination of piperine and chlorogenic acid. Interestingly, **3a** showed high cytotoxic activity on SK-MEL-147 at low concentration without affecting normal cells. Therefore, our data indicate that **3a** reduced viability of SK-MEL-147 cells with high potency and selectivity compared to its original prototypes. Thus, compound **3a** was selected for further investigations.

Antiproliferative activity of **3a** on SK-MEL-147 cells was characterized through different assays. Compound **3a** inhibited the clonogenic capacity of the SK-MEL-147 cells and induced cell cycle arrest. There was an augmentation of the G2/M population and a reduction in the frequency of cells at G1 and S. This result was consistent with a reduced frequency of cells labelled with the specific S-phase marker BrdU and an accumulation of cells in pro-metaphase and metaphase showing an aberrant mitotic spindle.

Compound **3a** downregulated the expression of the essential genes for G2/M transition and mitosis onset *CDK1* and *CCNB1* (cyclin B) [31,50]. **3a** also reduced the expression of *AURKA* (Aurora A)*, AURKB* (Aurora B)*,* and polo-like kinase (*PLK1*)*,* which are associated with the activation of several proteins during G2/M transition and mitosis progression, including CDK1-CCNB complexes. Studies have shown that increased G2/M population and apoptosis induction in tumor cells are associated with response to Aurora kinase or PLK1 inhibition [51,52], which is in accordance with our results. Therefore, it can be assumed that antiproliferative activity of **3a** on SK-MEL-147 is closely associated with its ability to modulate critical regulators of G2/M transition and mitosis onset [29]. Since AURK A/B and PLK1 are overexpressed in many cancer types, including melanoma, and considering that these serine/threonine mitotic kinases are currently regarded as attractive anticancer targets [51,53,54], **3a** represents a promising pre-clinical drug prototype.

The Aurora family is a group of serine/threonine protein kinases crucial for cell cycle control. AURKA drives centrosome maturation, centrosome separation and bipolar spindle assembly [55]. The inhibition of AURKA leads to chromosome segregation defects and abnormal mitotic spindles followed by aneuploidy and apoptosis [32,56]. AURKB is a catalytic subunit of chromosomal passenger complex, which is involved in the regulation of microtubule-kinetochore attachments, chromosome segregation and cytokinesis [32,57]. The inhibition of AURKB causes deficiency in chromosome bi-orientation and cytokinesis failure, leading to polyploidization and cell death [32]. PLK1 acts cooperatively with AURKA/B throughout division stages [28,30,32]. It is important to promote AURKA activation that, in turn, contributes for CDK1-CCNB activation [58].

Overexpression of the AURKA/B and PLK1 in melanoma has been correlated with poorer survival prospects [53,54,59]. The expression levels of Aurora kinases and related proteins were inversely correlated with immune infiltration and response of melanoma cells to immunotherapy [53]. In particular, AURKA overexpression in melanoma has been associated with gene amplification and copy number gain, although transcription activation and post-transcriptional mechanisms can increase AURKA expression even in the absence of gene amplification [54,60]. Moreover, positive correlation was observed between AURKA-positive tumors and *BRAF*^V600E^ mutation, suggesting that activation of MAPK signaling by *BRAF/NRAS* mutations impacts on AURKA expression [54]. In this way, pharmacological inhibition of AURKA by MLN8237 (alisertib) resulted in the reduction of melanoma cell proliferation and induced senescence and apoptosis in cells with *BRAF* and *NRAS* mutation, and in vemurafenib (BRAF inhibitor) resistant cells [54]. Pathria et al. [61] demonstrated that concomitant inhibition of AURKA/MEK might overcome resistance in *NRAS*-mutated melanomas, and Posch et al. [62] reported that combination of MEK and PLK1 inhibitors suppresses proliferation of *NRAS* mutant melanoma cells in vitro and in vivo. In addition, it was revealed that PLK1 and NOTCH inhibitors had synergistic antiproliferative effect on multiple human melanoma cells [63].

Compound **3a** downregulated *AURKA/B* and *PLK1*, which are transcriptionally regulated by FOXM1 [33]. This transcription factor is overexpressed in most cancers, and it has been associated with all hallmarks of cancer by regulating the expression of several cell cycle regulators [64]. Since overexpression of FOXM1 has been reported in melanoma, compounds that are able to inhibit FOXM1 and its target genes may be valuable as anticancer agents. We further demonstrated that **3a** was effective for reducing *FOXM1* expression at the transcriptional level in SK-MEL-147 cells, reinforcing the antitumor potential of this compound. It has been suggested that the combined inhibition of AURKA and FOXM1 could be considered a good strategy for patients with melanoma who are refractory to the combined use of BRAF/MEK inhibitors [54]. We demonstrated that **3a** was effective at reducing *FOXM1* expression at the transcriptional level in SK-MEL-147 cells, which reinforces the antitumor potential of this compound.

The expression of genes that control the final stages of the cell cycle, such as *AURKA/B* and *PLK1*, depends on the dissociation of BMYB from MuvB [39]. MuvB then associates with FOXM1, which activates genes that complete the G2/M phase. It has been shown that a delay in cell cycle progression can be caused by decreased activity of BMYB, FOXM1 or MuvB proteins [38]. In addition to reduced expression of late cell cycle genes, we also observed a cell cycle arrest in the G2/M, indicating that the cell does not complete the cell cycle. Molecular docking analysis showed that **3a** could bind to the CUL1-RBX1 complex responsible for degrading BMYB [40]. Thus, it might be suggested that **3a** could interfere with cell cycle by binding to the complex responsible for BMYB degradation, preventing its degradation and consequently preventing the activation of FOXM1 to express genes that conclude the G2/M phase.

Moreover, compound **3a** upregulated *CDKN1A* (p21), an important negative regulator of the cell cycle, which is mainly regulated by p53 at transcriptional levels [65]. Importantly, immunodetection of p21 evidenced its nuclear localization in SK-MEL-147 after treatment with **3a**. It is well known that p21 may act as oncogenic or suppressor factor depending on the cellular context [65]. Many other studies have revealed upregulation of p21 in response to aurora kinases and PLK1 inhibitors [52,66]. In addition, the oncogenic role of FOXM1 is also associated with its ability to induce degradation of p21 and other cyclin-dependent cyclin inhibitors (CKIs 21) [67]. We demonstrated that the augmentation of p21 promoted by **3a** was critical for its inhibitory effect on proliferation of SK-MEL-147 melanoma cells.

Despite the important role of p21 for checkpoint activation and inhibition of cell cycle, this protein also participates in other biological processes, such as DNA replication and repair, transcription, and apoptosis cell death [50]. Thus, we investigated the pro-apoptotic potential of **3a** on SK-MEL-147 cells. We observed a significant increase in the frequency of annexin V-positive cells, followed by an increase in the expression of the pro-apoptotic gene *BAX*, and a decrease in the expression of the anti-apoptotic gene *BCL2* indicating that there was activation of the intrinsic apoptosis pathway [68].

Finally, we investigated whether the ability of **3a** to promote apoptosis in SK-MEL-147 was associated with DNA damage induction and/or oxidative stress. However, we did not observe DNA damage in SK-MEL-147 cells treated with **3a** using the comet assay. Alternatively, we evaluated the expression profile of genes associated with the DNA damage signaling pathway (checkpoint kinase 2, *CHK2*) and oxidative stress response (*NFE2L2*, also known as *NRF2*) [36,37], but consistent with previous results, we also did not observe alteration in the expression of *NFE2L2*, while *CHK2* was downregulated. Taken together, our results suggest that cellular stress caused by mitosis arrest due to modulation of mitotic kinases represents the main mechanism associated with pro-apoptotic activity of **3a** on SK-MEL-147 melanoma cells. However, further investigation should be performed to better characterize at molecular level the events associated with ability of **3a** to induce cell death.

## 4. Materials and Methods

### 4.1. Chemistry

#### 4.1.1. Generalities

The Infrared (IR) spectra were generated in a Thermo Scientific USA (Nicolet iS50 model) IR spectrometer (Thermo Scientific, Wilmington, DE, USA), coupled to Pike Gladi ATR technologies. Nuclear Magnetic Resonance (NMR) spectra of ^1^H and ^13^C were obtained on a Bruker AC-300 spectrometer (Bruker, Billerica, MA, USA) operating at 300 MHz for ^1^H NMR and 75 MHz for ^13^C NMR. The samples were solubilized in deuterated methanol or pyridine using tetramethylsilane as the internal reference. Mass spectrometric analyses were performed in a Bruker Compass mass spectrometer by electrospray ionization (range of *m*/*z* of 80–1000). The purity of all synthetic compounds was determined by High-Performance Liquid Chromatography (HPLC) using Shimadzu equipment. Thin-layer chromatography experiments were performed on silica gel sheets 60 F254 (Merck, Darmstadt, Germany), and purification by chromatography column was performed on flash silica gel (Sigma-Aldrich, Saint Louis, MO, USA, 220–440 mesh, 0.035 mm–0.075 mm). The visualization of the substances in TLC experiments was performed by using an iodochloroplatinate reagent or in a UV chamber (λ = 254 or 365 nm). Melting points were measured without correction using Mars equipment (PFM II) with a crushed sample packaged in a capillary tube.

#### 4.1.2. Synthesis of the Target Compounds

##### General Method for the Preparation of the Hydrazides **5a–i**

To a solution of 1.0 molar eq. of the corresponding cinnamic acid (**4a–i**) in acetonitrile (10 mL) was added 1.2 eq of HOBt and 1.2 eq. of EDAC, keeping the reaction mixture under magnetic stirring and room temperature for 2 h. In a second reaction flask, a solution of hydrazine monohydrate (10 eq.) in 10 mL of acetonitrile was prepared at 0 °C and then added drop-by-drop to the first reaction mixture [69]. At the end of reaction (TLC), the reaction mixture was concentrated under low pressure, followed by the addition of an aqueous solution of 5% NaHCO_3_ (4 mL) to form a precipitate that was filtered to furnish the desired hydrazide derivatives **5a–i** in 28–87% yield.

##### General Method for the Preparation of the Target Derivatives **3a–i**

To a solution of piperonal (0.2 g, 1 eq.) in dry EtOH (5 mL) was added 6 drops of concentrated HCl, followed by a drop-by-drop addition of a solution of the corresponding hydrazide (**5a–i**, 1 eq.) in dry EtOH (5 mL). The reaction mixture was kept under stirring and room temperature up to total consumption of hydrazides (TLC). Finally, a precipitate was removed by filtration and washed with cold EtOH, resulting in the desired derivatives **3a–i** in 35–91% yield.

*(2E,10E)-N′-((benzo[d][1,3]dioxol-6-yl)methylene)-3-(4-hydroxy-3-methoxyphenyl)acrylohydrazide* (PQM-277, **3a**). Yellow solid (yield 68%), m.p. 138-143 °C. IR (ATR): ν 3524, 3432, 3043, 1668, 1624, 1592 and 1266 cm^−1^. ^1^H NMR (300 MHz, Pyridine-d_6_) δ 12.39 (d, *J* = 31.43 Hz 1H, CONHN), 8.68 (s, 1H, NCH), 7.99 (d, *J* = 15.97 Hz, HC-CH) 7.54 (s, 1H, Ar-H), 5.94 (d, *J =* 22.53 Hz, 2H, HC-CH and Ar-H), 5.17 (s, 2H, COH_2_OC) and 3.69 (d, *J =* 21.29 Hz, 3H, OCH_3_). ^13^C NMR (75 MHz, Pyridine-d_6_) δ 167.5, 148.6, 146.2, 143.5, 142.5, 142.1, 129.5, 126.9, 117.2, 116.7, 114.6, 112.3, 111.1, 108.5, 105.7, 101.8 and 55.4. HR-MS (ESI) *m*/*z*: Calcd for C_18_H_16_N_2_NaO_5_ [M+Na]^+^: 363.0957, found: 363.0956.

*(2E,10E)-N′-((benzo[d][1,3]dioxol-6-yl)methylene)-3-(2-hydroxyphenyl)acrylohydrazide* (PQM-279, **3b**). Yellow solid (yield 76%), m.p. 233–236 °C. IR (ATR): ν 3627, 3366, 3051, 1654, 1629, 1579 and 1255 cm^−1^. ^1^H NMR (300 MHz, Pyridine-d_6_) δ 12.46 (d, *J* = 77.63 Hz, 1H, CONHN), 8.97 (d, *J =* 16.00 Hz, 1H, OH), 8.68 (s, 1H, NCH), 7.75 (d, *J =* 7.24 Hz, 1H, HC-CH), 7.45 (d, *J =* 17.08 Hz, 1H, Ar-H), 5.93 (d, *J =* 22.78 Hz, 2H, HC-CH and Ar-H) and 5.19 (s, 2H, COH_2_OC). ^13^C NMR (75 MHz, Pyridine-d_6_) δ 168.0, 158.3, 148.6, 146.3, 142.2, 138.9, 138.2, 131.3, 129.7, 129.2, 126.6, 119.7, 117.6, 116.7, 108.4, 105.9, and 101.8. HR-MS (ESI) *m*/*z*: Calcd for C_17_H_15_N_2_O_4_ [M+H]^+^: 311.1032, found: 311.1079.

*(2E,10E)-N′-((benzo[d][1,3]dioxol-6-yl)methylene)-3-(4-hydroxyphenyl)acrylohydrazide* (PQM-280, **3c**). Yellow solid (yield 35%), m.p. > 300 °C. IR (ATR): ν 3185, 3063, 1639, 1585 and 1282 cm^−1^. ^1^H NMR (300 MHz, Pyridine-d_6_) δ 12.38 (d, *J =* 41.83 Hz, 1H, CONHN), 8,79 (s, 1H, OH), 8.68 (s, 1H, NCH), 7.95 (d, *J =* 15.86 Hz, 1H, HC-CH)), 6.79 (d, *J =* 7.85 Hz, 1H, HC-CH), 5.93 (d, *J =* 22.75 Hz, 2H, Ar-H) and 5.20 (s, 2H, COH_2_OC). ^13^C NMR (75 MHz, Pyridine-d_6_) δ 167.5, 160.8, 148.6, 146.3, 143.1, 142.5, 141.9, 130.5, 130.0, 129.8, 129.6, 116.9, 114.3 and 101.8. HR-MS (ESI) *m*/*z*: Calcd for C_17_H_14_N_2_NaO_4_ [M+Na]^+^: 333.0852, found: 333.0839.

*(2E,10E)-N′-((benzo[d][1,3]dioxol-6-yl)methylene)cinnamohydrazide* (PQM-281, **3d**). Yellow solid (yield 42%), m.p. 198–203 °C. IR (ATR): ν 3339, 3001, 1665, 1593 and 1259 cm^−1^. ^1^H NMR (300 MHz, Pyridine-d_6_) δ 12.50 (d, *J* = 33.53 Hz, 1H, CONHN), 8.64 (s, 1H, NCH), 7.24 (m, 5H, Ar-H) 6.90 (d, *J =* 7.97, 1H, HC-CH), 5.94 (d, *J =* 25.03 Hz, 2H, HC-CH and Ar-H)) and 5.19 (s, 2H, COH_2_OC). ^13^C NMR (75 MHz, Pyridine-d_6_) δ 166.9, 148.6, 146.9, 143.0, 142.6, 141.4, 129.9, 128.4, 127.9, 120.7, 118.3, 108.5, 105.7 and 101.9. HR-MS (ESI) *m*/*z*: Calcd for C_17_H_14_N_2_NaO_3_ [M+Na]^+^: 317.0902, found: 317.0881.

*(2E,10E)-3-(benzo[d][1,3]dioxol-6-yl)-N′-((benzo[d][1,3]dioxol-6-yl)methylene)acrylohydrazide* (PQM-284, **3e**). Yellow solid (yield 31%), m.p. 208–210 °C. IR (ATR): ν 3586, 3042, 1662, 1619, 1563 and 1212 cm^−1^. ^1^H NMR (300 MHz, Pyridine-d_6_) δ 12.32 (s, 1H, CONHN), 8.68 (s, 1H, NCH), 7.93 (d, *J =* 15.81, 1H, HC-CH), 7.41 (s, 1H, Ar-H) and 5.00 (s, 4H, COH_2_OC). ^13^C NMR (75 MHz, Pyridine-d_6_) δ 167.2, 148.6, 147.7, 146.9, 142.6, 141.3, 130.1, 129.8, 129.5, 124.5, 118.6, 116.5, 108.6, 108.5, 107.2, 106.4, 105.8 and 101.8. HR-MS (ESI) *m*/*z*: Calcd for C_18_H_14_N_2_NaO_5_ [M+Na]^+^: 361.0801, found: 361.0709.

*(2E,10E)-N′-((benzo[d][1,3]dioxol-6-yl)methylene)-3-(4-(trifluoromethyl)phenyl)acrylohydrazide* (PQM-285, **3f**). White solid (yield 91%), m.p. 237–245 °C. IR (ATR): ν 3383, 2988, 1666, 1621, 1588, 1325 and 1257 cm^−1^. ^1^H NMR (300 MHz, Pyridine-d_6_) δ 12.51 (s, 1H, CONHN), 8.68 (1H, NCH), 7.76 (d, *J =* 8.16 Hz, 1H, HC-CH), 7.53 (s, 1H, Ar-H), 6.91 (d, *J =* 8.00 Hz, 1H, Ar-H), 6.82 (d, *J =* 7.95 Hz, 1H, Ar-H), 5.95 (d, *J =* 22.58 Hz, 2H, Ar-H and HC-CH) and 4.94 (s, 2H, COH_2_OC). ^13^C NMR (75 MHz, Pyridine-d_6_) δ 166.4, 148.7, 147.3, 143.2, 140.6, 139.5, 128.7, 128.2, 125.6, 121.0, 108.5, 105.7 and 101.9. HR-MS (ESI) *m*/*z*: Calcd for C_18_H_14_N_2_NaO_5_ [M+Na]^+^: 385.0776, found: 385.0764.

*(2E,10E)-N′-((benzo[d][1,3]dioxol-6-yl)methylene)-3-(2-hydroxy-3-methoxyphenyl)acrylohydrazide* (PQM-286, **3g**). Yellow solid (yield 85%), m.p. 277–279 °C. IR (ATR): ν 3228, 3058, 2899, 1654, 1622, 1545 and 1248 cm^−1^. ^1^H NMR (300 MHz, Pyridine-d_6_) δ 12.26 (d, *J =* 19.30 Hz, 1H, CONHN), 11.34 (s, 1H, OH), 8.68 (s, 1H, NCH), 7.96 (d, *J =* 15.95 Hz, 1H, HC-CH), 7.58 (m, 3H, Ar-H), 5.94 (d, *J =* 27.12 Hz, 2H, HC-CH and Ar-H), 4.96 (s, 2H, COH_2_OC) and 3.69 (d, *J =* 11.65, 3H, OCH_3_). ^13^C NMR (75 MHz, Pyridine-d_6_) δ 167.3, 148.6, 148.3, 146.4, 143.2, 142.5, 141.9, 129.8, 121.2, 120.8, 118.0, 115.43, 115.2, 114.6, 112.0, 108.5, 105.7, 101.8 and 55.6. HR-MS (ESI) *m*/*z*: Calcd for C_18_H_16_N_2_NaO_5_ [M+Na]^+^: 363.0957, found: 363.0954.

*(2E,10E)-N′-((benzo[d][1,3]dioxol-6-yl)methylene)-3-(2,3-dimethoxyphenyl)acrylohydrazide* (PQM-287, **3h**). Yellow solid (yield 89%), m.p. 239–243 °C. IR (ATR): ν 3132, 3064, 1665, 1622, 1509 and 1257 cm^−1^. ^1^H NMR (300 MHz, Pyridine-d_6_) δ 12.44 (d, *J* = 36.18 Hz, 1H, CONHN), 8.68 (s, 1H, NCH), 7.96 (d, *J* = 15.84 Hz, 1H, HC-CH), 7.54 (s, 1H, Ar-H), 7.47 (d, *J* = 8.40 Hz, 1H, Ar-H), 5.95 (d, *J* = 24.76 Hz, 2H, HC-CH and Ar-H), 5.19 (s, 2H, COH_2_OC) and 3.62 (d, *J* = 10.95 Hz, 3H, OCH_3_). ^13^C NMR (75 MHz, Pyridine-d_6_) δ 167.3, 148.6, 146.5, 142.7, 142.5, 141.3, 130.1, 129.6, 128.3, 118.5, 118.2, 115.5, 114.6, 108.3, 105.9, 105.7 and 101.9. HR-MS (ESI) *m*/*z*: Calcd for C_19_H_19_N_2_O_5_ [M+H]^+^: 355.1294, found: 355.1210.

*(2E,10E)-N′-((benzo[d][1,3]dioxol-6-yl)methylene)-3-(4-chlorophenyl)acrylohydrazide* (PQM-288, **3i**). Yellow solid (Yield 85%), m.p. 238–243 °C. IR (ATR): ν 3385, 2994, 1663, 1619, 1589 and 1259 cm^−1^. ^1^H NMR (300 MHz, Pyridine-d_6_) δ 12.42 (s, 1H, CONHN), 8.68 (s, 1H, NCH), 7.53 (s, 1H, Ar-H), 7.36 (d, *J =* 8.38 and 15.19 Hz, 2H, Ar-H), 6.81 (d, *J =* 8.00 Hz, 1H, HC-CH), 5.94 (d, *J =* 22.86 Hz, 2H, HC-CH and Ar-H) and 4.96 (s, 2H, COH_2_OC). ^13^C NMR (75 MHz, Pyridine-d_6_) δ 166.9, 148.7, 147.9, 143.0, 141.1, 139.9, 134.4, 129.8, 129.3, 121.4, 118.9, 108.5, 105.8 and 101.8. HR-MS (ESI) *m*/*z*: Calcd for C_17_H_13_ClN_2_NaO_3_ [M+Na]^+^: 351.0513 found: 351.0492.

### 4.2. Cell Lines, Culture Cell Conditions, and Treatment Schedule

Melanoma cell lines (SKMEL-147, CHL-1 e WM1366) and primary dermal fibroblast cell culture (PDF) were used in this study. The cells were maintained in DMEM/F12 (Dulbecco’s Modified Eagle’s Medium plus F12, Sigma-Aldrich) supplemented with 10% fetal bovine serum (Vitrocell, Campinas, Brazil). Cells were grown in a humidified atmosphere of 95% air and 5% CO_2_ at 37 °C.

Synthetic compounds were solubilized in dimethyl sulfoxide (DMSO), and stock solution (20 mM) was stored at −20 °C until use. The substances were solubilized in fresh culture medium at final concentrations immediately before treatments. Cells were seeded into plates containing 96, 24, or 6 wells, depending on the experimental approach. After attachment, the cells were treated for 24 or 48 h.

### 4.3. Cell Viability Assay

Cell viability analysis was performed according to Vichai and Kirtikara [70]. The sulforhodamine B (SRB) assay was used for cell density determination, based on the measurement of cellular protein content. Melanoma cells were seeded into 96-well plates at a density of 1 × 10^4^ per well. After attachment (24 h), the cells were treated with different substances at 25 μM for 48 h. Cell monolayers were fixed with 10% (wt/vol) trichloroacetic acid at 4 °C for 1h and stained with SRB (0.4% in 1% acetic acid) for 30 min. Next, the samples were washed repeatedly with 1% (vol/vol) acetic acid to remove unbound SRB. The protein-bound dye was dissolved in 10 mM Tris-base solution for optical density (OD) determination at 540 nm with reference of 690 nm, using a microplate reader.

### 4.4. Cell Cycle Analysis

Cell cycle analysis was performed according to Ferreira-Silva et al. [71]. Briefly, SK-MEL-147 cells were seeded into 35 mm Petri plates at a density of 1 × 10^5^ cells/plate. Cell cultures were treated with **3a** at 5 or 10 μM for 24 h. Then, the samples were fixed with 75% ethanol at 4 °C overnight, and rinsed twice with ice-cold phosphate-buffered saline (PBS). Afterwards, cells were homogenized in dye solution [PBS containing 90 μg·mL^−1^ propidium iodide (PI) and 1.5 mg·mL^−1^ RNAase]. The analysis was performed using a flow cytometer (Guava easyCyte 8HT, Hayward, CA, USA).

### 4.5. Clonogenic Assay

Cells were seeded into 35 mm plates (200 cells/plate). After adherence (24 h), the cells were treated with **3a** at 5–10 μM for 24 h and recovered in a drug-free medium for subsequent 12 days. Afterwards, the colonies were fixed with methanol and stained with crystal violet. Only colonies with >50 cells were counted by direct visual inspection with a stereomicroscope at 20× magnification.

### 4.6. Mitotic Index Determination

Mitotic indices were determined as previously described by Azevedo-Barbosa et al. [72]. Briefly, cells were plated in 35 mm Petri dishes (1 × 10^5^ cells) and samples were treated with **3a** at 5 and 10 μM for 24 h. Fluorescent cytological preparations evidencing microtubule network and nuclei were used to determine the frequency of mitosis (1000 cells per sample were counted).

### 4.7. BrdU Incorporation Assay

The frequency of cells in S-phase was determined by bromodeoxyuridine (BrdU) incorporation assay. First, 1 × 10^5^ cells were seeded into 35 mm Petri dishes and samples were treated with **3a** for 24 h at 5–10 μM. Two hours before the end of the treatment, a BrdU (CAS-number 59–14-3) pulse (100 µM) was performed. After that, BrdU immunodetection was carried out. Cells were fixed in 3.7% formaldehyde (15 min) at room temperature, and DNA was denatured using 1.5 mol L^−1^ of HCl (30 min). After washing, the samples were incubated with anti-BrdU antibody (Cell signaling, #5292, 1:500) overnight at 4 °C. Afterward, anti-mouse secondary antibody-FITC conjugated (1:100, Sigma) was added to the samples (2 h at room temperature), followed by DAPI staining (1 h at room temperature) (#D3571, ThermoFisher, Waltham, MA, USA). Cytological preparations were analyzed using a fluorescence microscope (Nikon, Tokyo, Japan, eclipse 80i).

### 4.8. Apoptosis Detection by Annexin V/7-AAD

Guava Nexin^®^ kit (Merk Millipore, Massachusetts, EUA) was used according to the manufacturer’s instructions to detect apoptosis. Briefly, 1 × 10^5^ cells/well were seeded into 24-well plates, and the samples were treated with **3a** at 5–10 μM or cisplatin at 20–40 μM for 24 or 48 h. The cells were collected using Tryipsin/EDTA solution (T4299, Sigma) and the samples were centrifuged at 200× *g* for 5 min at 4 °C. After washing with ice-cold PBS, 2 × 10^4^ cells were suspended in culture medium (100 μL) and Annexin V-PE/ 7-AAD mix solution (100 μL) was added. After 20 min of incubation at room temperature in a dark chamber, the samples were analyzed by flow cytometry.

### 4.9. Immunoblot

Cells were seeded into 100 mm Petri dishes at 1 × 10^6^ cells/plate. The samples were treated with **3a** at 5 and 10 μM for 24 or 48 h. Cellular homogenate was obtained using RIPA lysis buffer with protease and phosphatase inhibitors (Sigma, #P8340). After centrifugation (10,000× *g* for 10 min at 4 °C), supernatants were collected, and quantification of total proteins was determined using BCA kit (Pierce Biotechnology Inc., Waltham, MA, USA). 50 μg of total protein was separated by SDS–PAGE (12%) and transferred (100 V, 250 mA for 2 h) onto a PVDF membrane (Amersham Bioscience, Amersham, UK). After membrane blocking (5% non-fat milk in Tris-buffered saline + 0.1% Tween20) (1 h at 4 °C), it was probed with primary antibodies: anti-phospho-ERK(Tyr 204) (Santa-Cruz, sc-7383, 1:200), anti-ERK 1/2 (Cell signaling, #4696, 1:1000), anti-Cyclin B1 (Santa Cruz, sc-245, 1:200), anti-AKT(Ser 473) (Cell signaling, #4060), and α-tubulin (Sigma–1:1000) overnight at 4 °C. After washing, the membrane was incubated with an appropriate secondary antibody—HRP conjugated for 2h at room temperature. Immunoreactive bands were revealed by ECL Western blotting Detection Kit (Amersham Bioscience), and quantified by ImageJ [73].

### 4.10. Comet Assay

Alkaline comet assay was performed as described previously by [74] with some modifications. Briefly, cells were seeded in tissue culture flasks and incubated for 12 and 24 h with **3a** at 10 μM. After checking the cell viability rate (at least 70% of viable cells), two hundred thousand cells were suspended in 0.5% low melting point agarose (Sigma-Aldrich). Cellular suspension was spread into microscope slides pre-coated with 1.5% normal-melting point agarose (Sigma-Aldrich). After lysis, electrophoresis was performed. Finally, the samples were neutralized, fixed, and stained with SYBR^®^ Green I solution (Invitrogen by Thermo Fisher Scientific Inc., Rockford, IL, USA). The analysis was performed using a fluorescence microscope at 20× magnification. The images were analyzed using the ImageJ [73] plugin OpenComet [75] to demarcate the “head” and the “tail” regions of each comet. Samples treated with ultraviolet light were used as a positive control. Fifty randomly selected nuclei were analyzed sample. The analysis was done by the extent of DNA damage (tail moment).

### 4.11. Transcript Level Evaluation

Real-time PCR was performed according to Lamartine-Hanemann et al. [76]. Briefly, the cells were seeded into 6-well plates at a density of 1 × 10^5^ cells per well. Total RNA was extracted using the RNeasy^®^ Mini kit (Qiagen, Mississauga, ON, Canada) and RNA concentrations were measured by spectrophotometer using a NanoDrop^®^ ND 1000 (Thermo Scientific, Wilmington, DE, USA). Then, 1 µg of total RNA was incubated with DNase I (1 U/µg; Invitrogen, São Paulo, SP, Brazil) and subjected to reverse transcription using random primers, and the High-Capacity cDNA Reverse Transcription Kit^®^ (Applied Biosystems, São Paulo, SP, Brazil).

Expression of the target genes (Table S1) was investigated by real-time PCR with an ABI 7500 thermocycler using Power Sybr Green PCR Master Mix (Applied Biosystems). The genes were amplified using the following conditions: 95 °C for 10 min (1 cycle), denaturation at 95 °C for 10 s, and annealing and extension at 60 °C for 1 min (40 cycles). To select the most stable housekeeping gene, β-actin (*ACTB*), glyceraldehyde-3- phosphate dehydrogenase (*GAPDH*), and 18S ribosomal RNA (*18srRNA*) amplification profiles were compared using the geNorm applet for Microsoft Excel [77]; the most stable housekeeping gene was ATCB. The relative abundance of each target gene was calculated using the ΔΔCt method with efficiency correction and a control sample for calibration [78]. Each sample was run in triplicate, and non-template control was included.

### 4.12. Molecular Docking

Computational docking was performed to check the interaction between **3a** and the CUL1-RBX1 SCF Ubiquitin Ligase Complex [79]. The crystal structure of the CUL1-RBX1 complex was obtained from RCSB Protein Data Bank (PDB) (rcsb.org, accessed on 15 July 2022) of PDB ID 1LDJ [79]. The **3a** ligand file and the receptor file were prepared using the AutoDockTools package version 1.5.7 [41] using default parameters. Docking was performed using a grid map dimension of 40 × 40 × 40 Å using default parameters with AutoDock Vina version 1.1.2 [41] and exhaustiveness at 24. Visualization and figure generation using the original structure of ID 1LDJ [79] was performed using Discovery Studio Visualizer v21.1.0.20298.

### 4.13. Statistical Analysis

The results were tested for significance using *t* test or one-way analysis of variance (ANOVA) followed by Dunnet’s post-test using GraphPad Prism^®^. *P* values < 0.05 were considered statistically significant. The results from qPCR are presented as standard error of the mean (SEM) of 4 independent experiments. Other results are corresponding to mean ± standard deviation (SD) from at least three independent experiments.

## 5. Conclusions

We designed and synthesized a series of nine hybrid compounds bearing piperine and chlorogenic acid fragments and evaluated their antitumor potential on melanoma cell lines with different mutation profiles. SK-MEL-147 was more sensitive to these compounds than CHL-1 or WM-1366 cells. The most cytotoxic compound was **3a**, which inhibited cell cycle progression and reduced the expression of *CDK1*, Auroras A/B, *PLK1*, that are required for G2/M transition and mitosis onset. Meanwhile, there was a significant upregulation of the cell cycle inhibitor p21. We provided insight that effects of **3a** on SK-MEL-147 could be mediated by *FOXM1*, a master regulator of mitotic gene expression, required for cell proliferation. Docking studies predicted that compound **3a** could interact with the CUL1 ubiquitin ligase complex responsible for BMYB degradation compromising the activation of FOXM1. The cellular stress caused by **3a** promoted cell cycle arrest and subsequently apoptosis by the intrinsic pathway. Our findings demonstrate that **3a** is an important antitumor candidate, and support further investigations to evaluate its potential for melanoma treatment, especially for refractory cases to BRAF/MEK inhibitors.

## Data Availability

Not applicable.

## Supplementary Materials

The following supporting information can be downloaded at: https://www.mdpi.com/article/10.3390/ph16020145/s1, Table S1: Sequences of the primers used for amplification in real-time PCR; Figures S1–S36: Infrared absorption spectrum (ATR), 1H spectrum (300 MHz, pyridine d5), 13C spectrum (75 MHz, pyridine d5) and Mass Spectrum (positive mode) of the compounds **3a–i**.
