# Supplementary material for: Piperine–Chlorogenic Acid Hybrid Inhibits the Proliferation of the SK-MEL-147 Melanoma Cells by Modulating Mitotic Kinases
Source: Pharmaceuticals (Basel). 2023 Jan 19;16(2):145. doi: 10.3390/ph16020145 (PMC9965075; doi:10.3390/ph16020145)
Supplement: Supplementary file 1 [file pharmaceuticals-16-00145-s001.zip › pharmaceuticals-2118160-supplementary.pdf]

Supplementary Material

Piperine–Chlorogenic Acid Hybrid Inhibits the Proliferation of the SK-MEL-147 Melanoma Cells by Modulating Mitotic Kinases

Carolina Girotto Pressete [1], Flávia Pereira Dias Viegas [2], Thâmara Gaspar Campos [2], Ester Siqueira Caixeta [1], João Adolfo Costa Hanemann [3], Guilherme Álvaro Ferreira-Silva [1], Bruno Zavan [1], Alexandre Ferro Aissa [1], Marta Miyazawa [3,*], Claudio Viegas Jr. [2,*] and Marisa Ionta [1,*]

[1] Institute of Biomedical Sciences, Federal University of Alfenas, Alfenas 37130-001, MG, Brazil
[2] Institute of Chemistry, Laboratory of Research in Medicinal Chemistry, Federal University of Alfenas, Alfenas 37133-840, MG, Brazil
[3] Department of Clinic and Surgery, School of Dentistry, Federal University of Alfenas, Alfenas 37130-001, MG, Brazil
* Correspondence: marta.miyazawa@unifal-mg.edu.br (M.M.); claudio.viegas@unifal-mg.edu.br (C.V.J.); marisa.ionta@unifal-mg.edu.br (M.I.)

Table S1. Sequences of the primers used for amplification in real-time PCR.

Gene	Sequence	Reference
CDKN1A	F 5'- CCATAGCCTCTACTGCCACCATC-3'	NM_001291549.1
	R 5'- GTCCAGCGACCTTCCTCATCCA-3'	
CCNB1	F 5'- GTACCCTCCAGAAATTGGTGA-3'	NM_031966.2
	R 5'- GACTACATTCTTAGCCAGGTG-3'	
NRF2	F 5'- CAATGAGGTTTCTTCGGCTACG -3'	NM_006164.4
	R 5'- AAGACTGGGCTCTCGATGTG -3'	
CHK2	F 5'- CCCAAGGCTCCTCCTCACA -3'	NM_007194.3
	R 5'- AGTGAGAGGACTGGCTGGAGTT -3'	
AURKA	F 5'- TCTTCACAGGAGGCAAATCCA-3'	NM_198434.3
	R 5'- AATAAGTTACACACTCACTCAGGTACTA-3'	
AURKB	F 5'- AAAGAGCCTGTCACCCCATC-3'	NM_001313950.2
	R 5'- CGCCCAATCTCAAAGTCATC-3'	
BAX	F:5'- TTCCTTACGTGTCTGATCAATCC-3'	NM_004324.3
	R:5'- GGGCAGAAGGCACTAATCAA -3'	
BCL-2	F:5'- CAGAAGTCTGGGAATCGATCTG -3'	NM_000657.2
	R:5'- AATCTTCAGCACTCTCCAGTTATAG -3'	
FOXM1	F: 5′-TGCCCAGCAGTCTCTTACCT-3′	NM_001243089.1
	R: 5′-CTACCCACCTTCTGGCAGTC-3′	
ACTB	F 5'- AGAGCTACGAGCTGCCTGAC-3'	NM_001101.3
	R 5'- AGCACTGTGTTGGCGTACAG-3'	
GAPDH	F 5'- GGATTTGGTCGTATTGGGC-3'	NM_002046.4
	R 5'- TGGAAGATGGTGATGGGATT-3'	
18srRNA	F 5'- GTAACCCGTTGAACCCCATT-3'	HQ387008.1

R 5’- CCATCCAATCGGTAGTAGCG-3’

F = forward primer; R = reverse primer

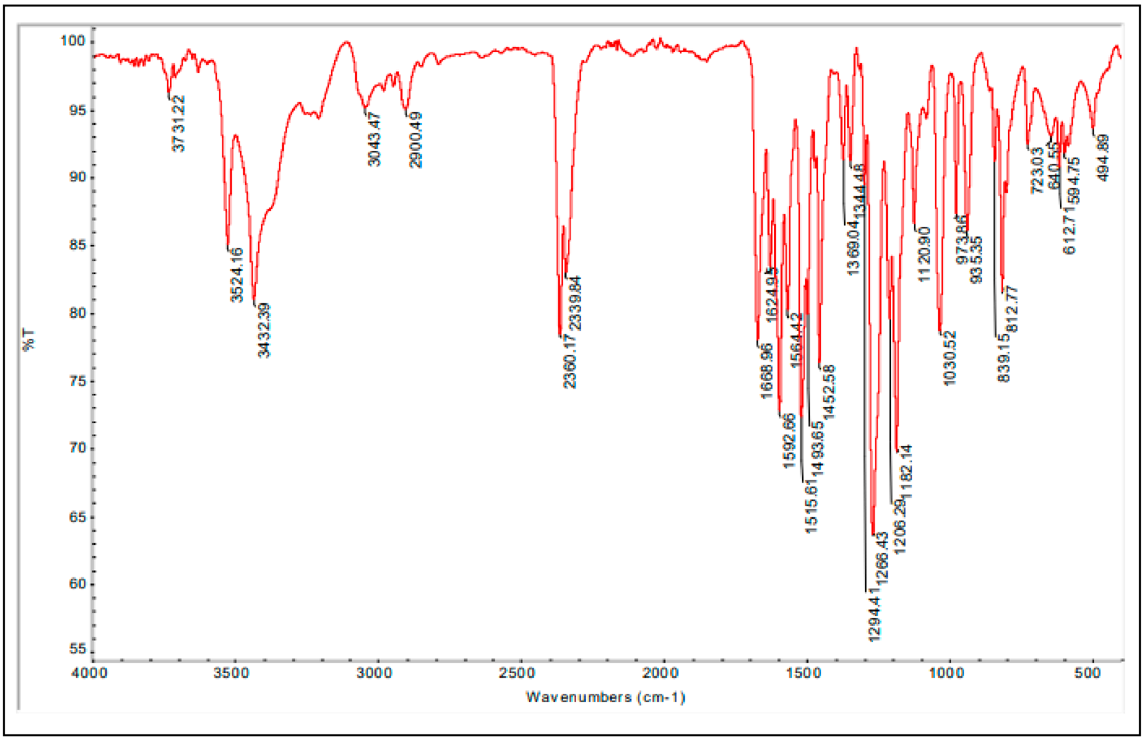

Figure S1 – Infrared absorption spectrum (ATR) of the compound **PQM-277 (3a)**.

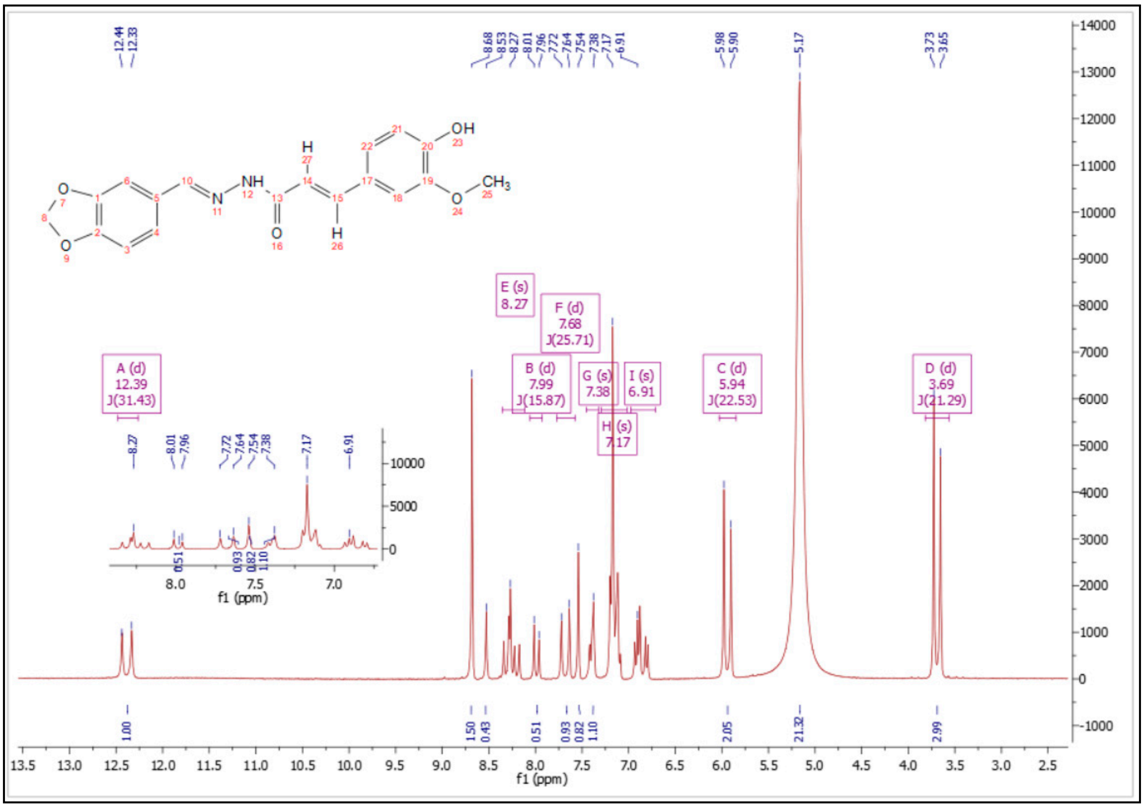

Figure S2 – ^{1}H spectrum (300 MHz, pyridine *d*5) of the compound **PQM-277 (3a)**.

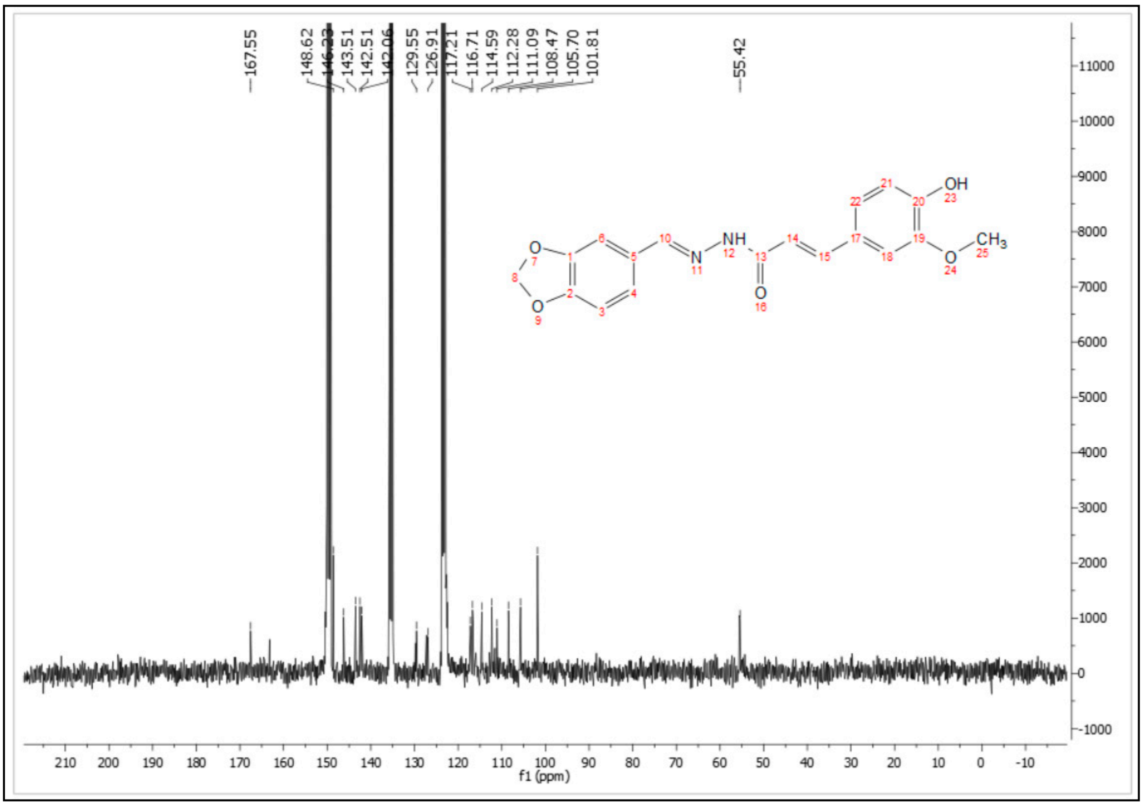

Figure S3 – ^{13}C spectrum (75 MHz, pyridine *d*5) of the compound **PQM-277 (3a)**.

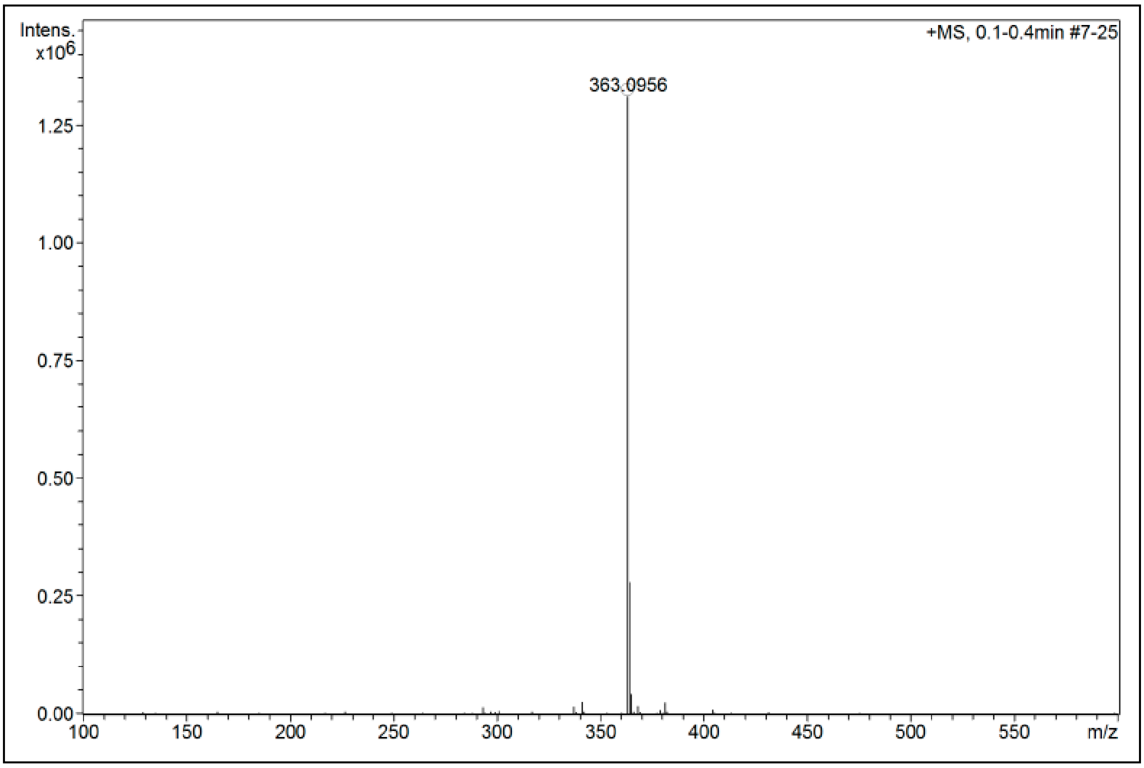

Figure S4 – Mass Spectrum (positive mode) of the compound **PQM-277 (3a)**.

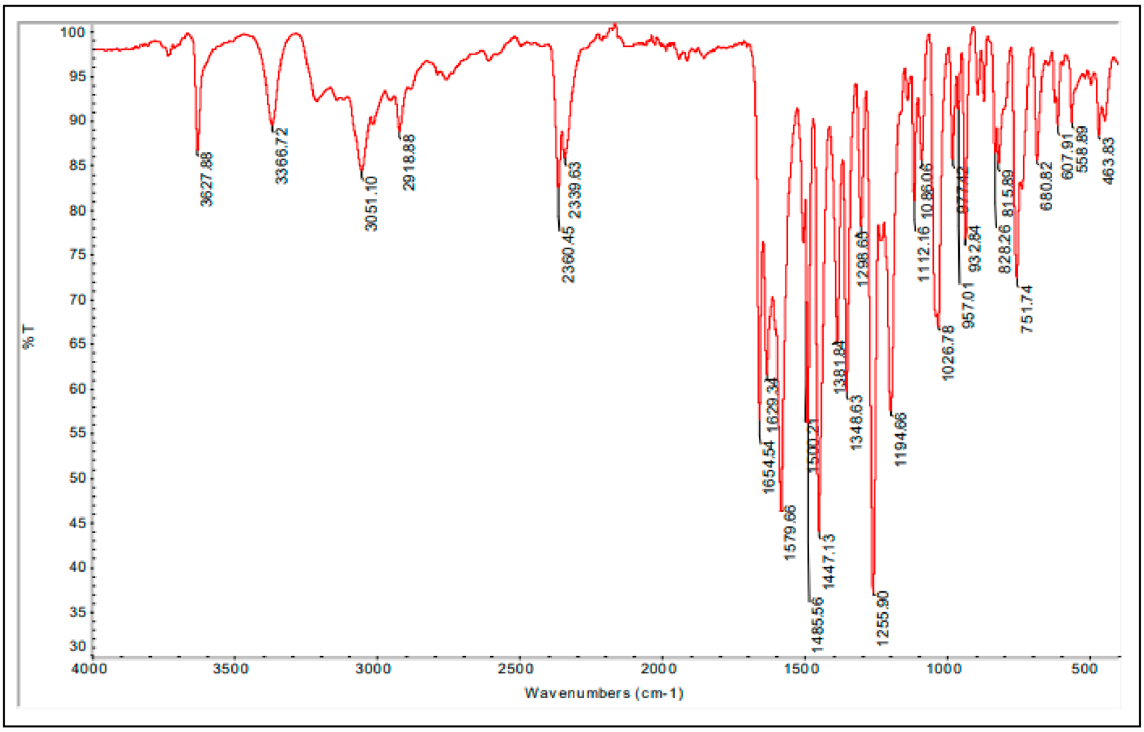

Figure S5 – Infrared absorption spectrum (ATR) of the compound **PQM-279 (3b)**.

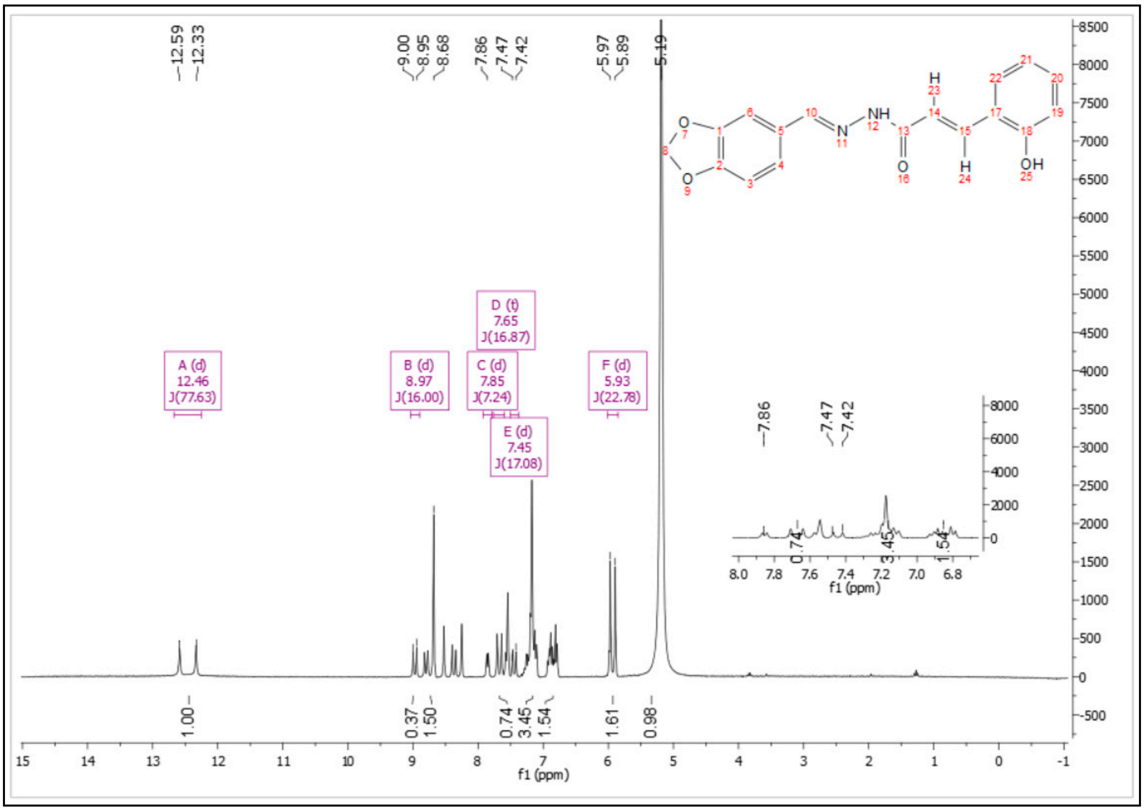

Figure S6 – ^{1}H spectrum (300 MHz, pyridine *d*5) of the compound **PQM-279 (3b)**.

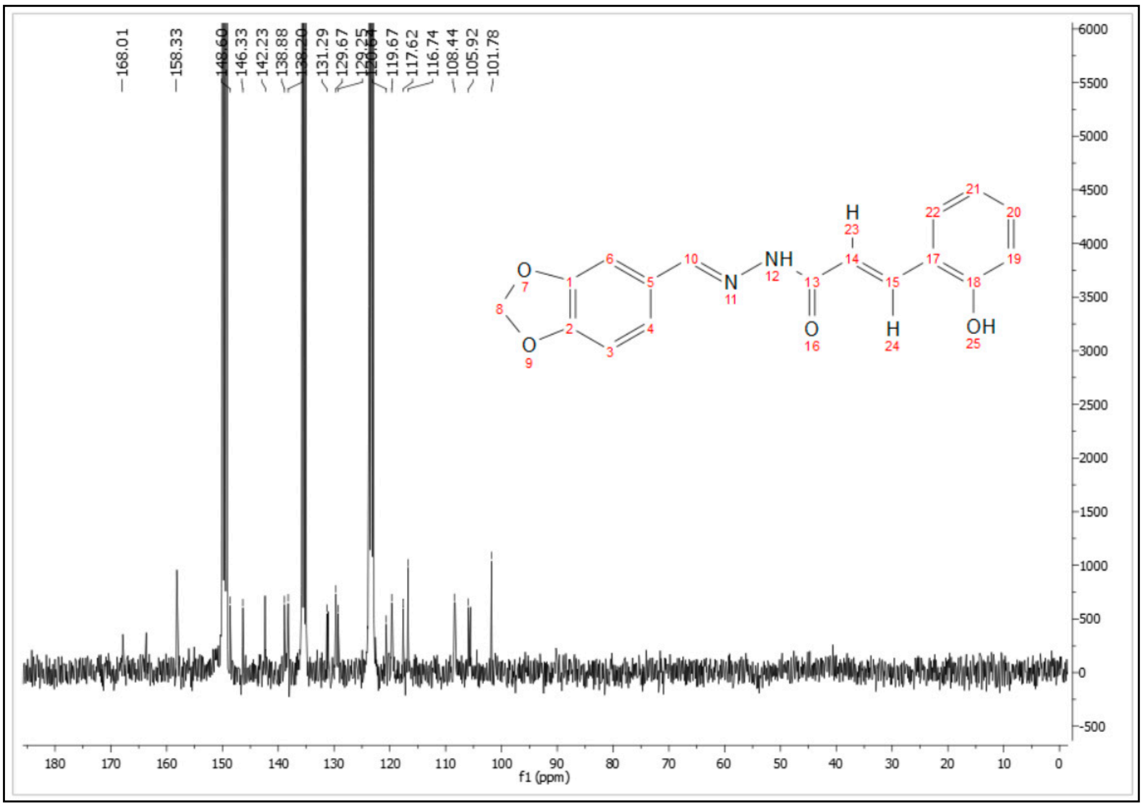

Figure S7 – ^{13}C spectrum (75 MHz, pyridine *d*5) of the compound **PQM-279 (3b)**.

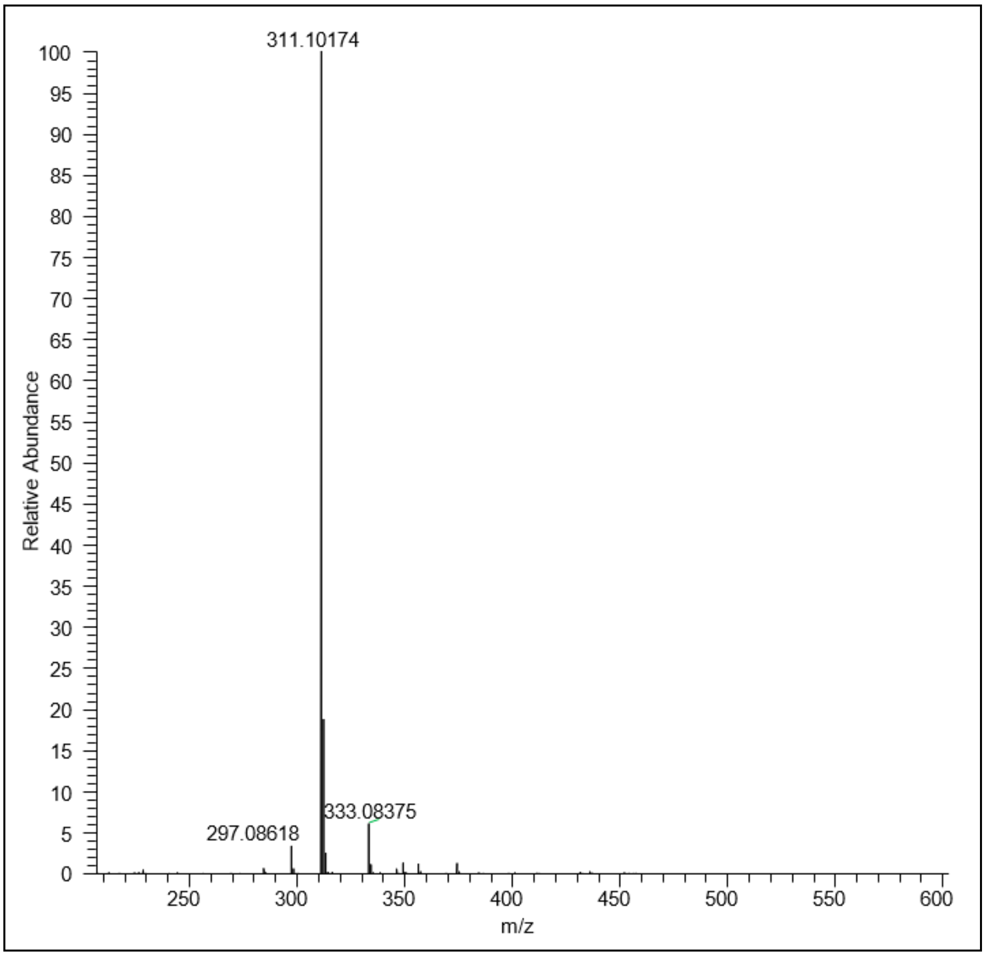

Figure S8 – Mass Spectrum (positive mode) of the compound **PQM-279 (3b)**.

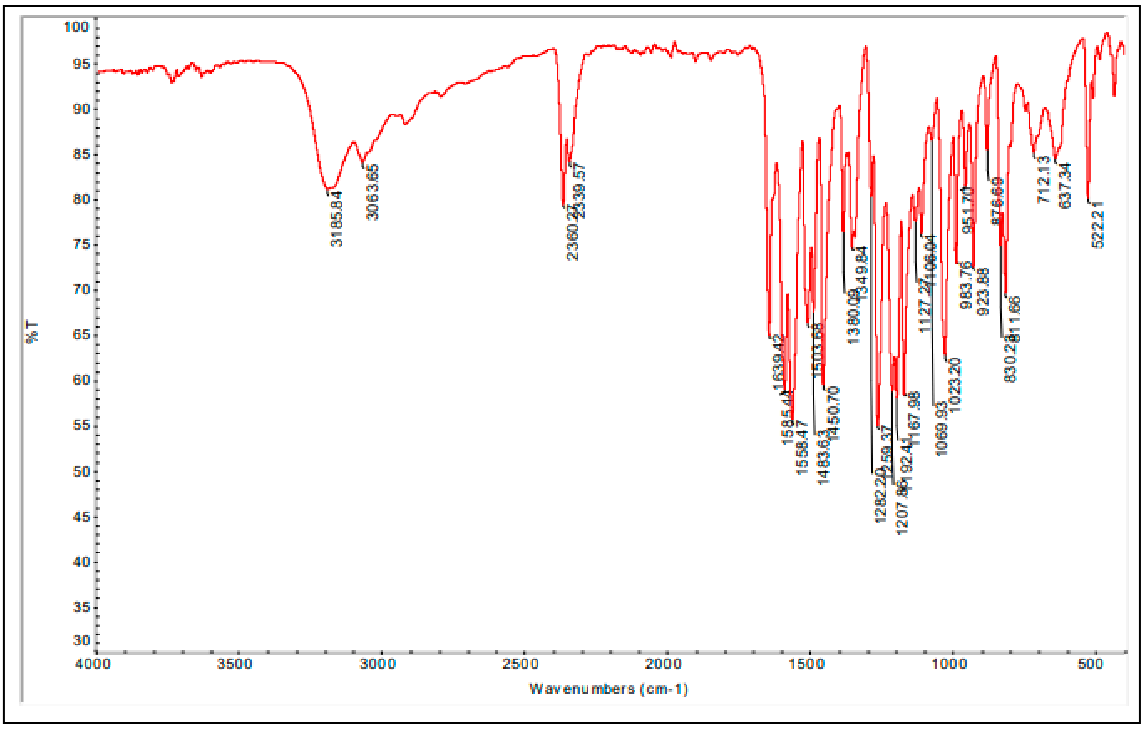

Figure S9 – Infrared absorption spectrum (ATR) of the compound **PQM-280 (3c)**.

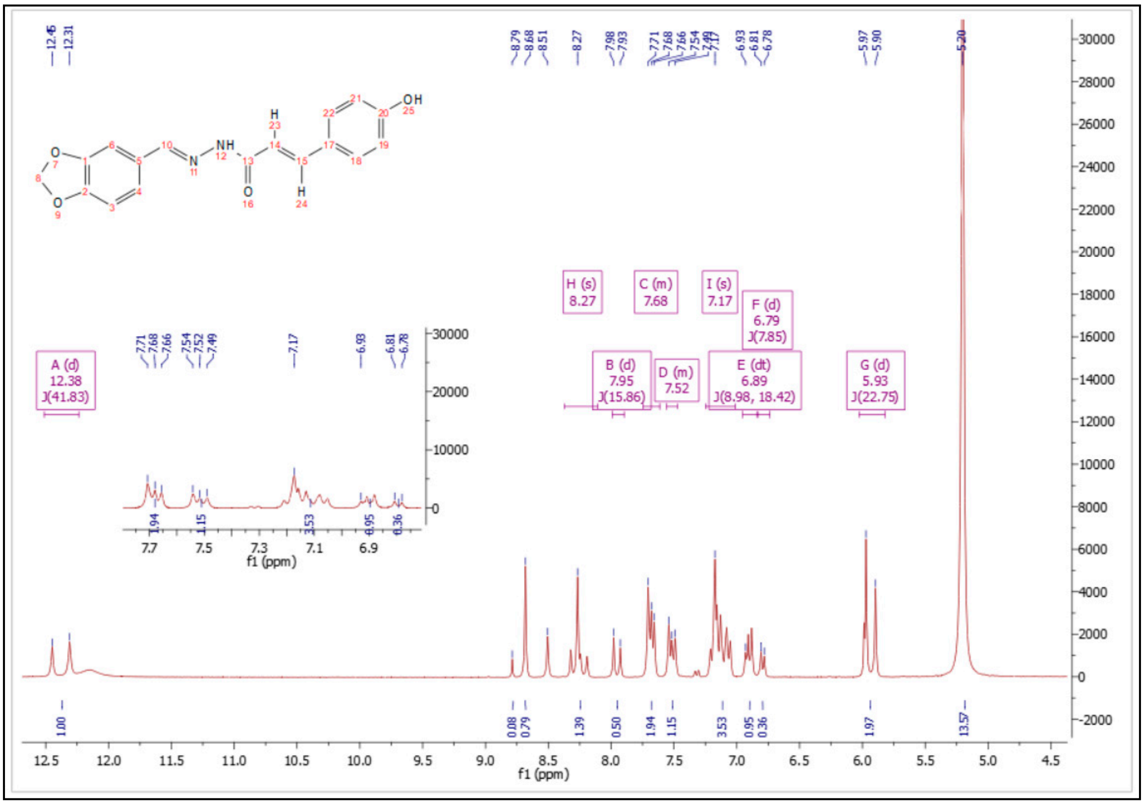

Figure S10 – ^{1}H spectrum (300 MHz, pyridine d_5) of the compound **PQM-280 (3c)**.

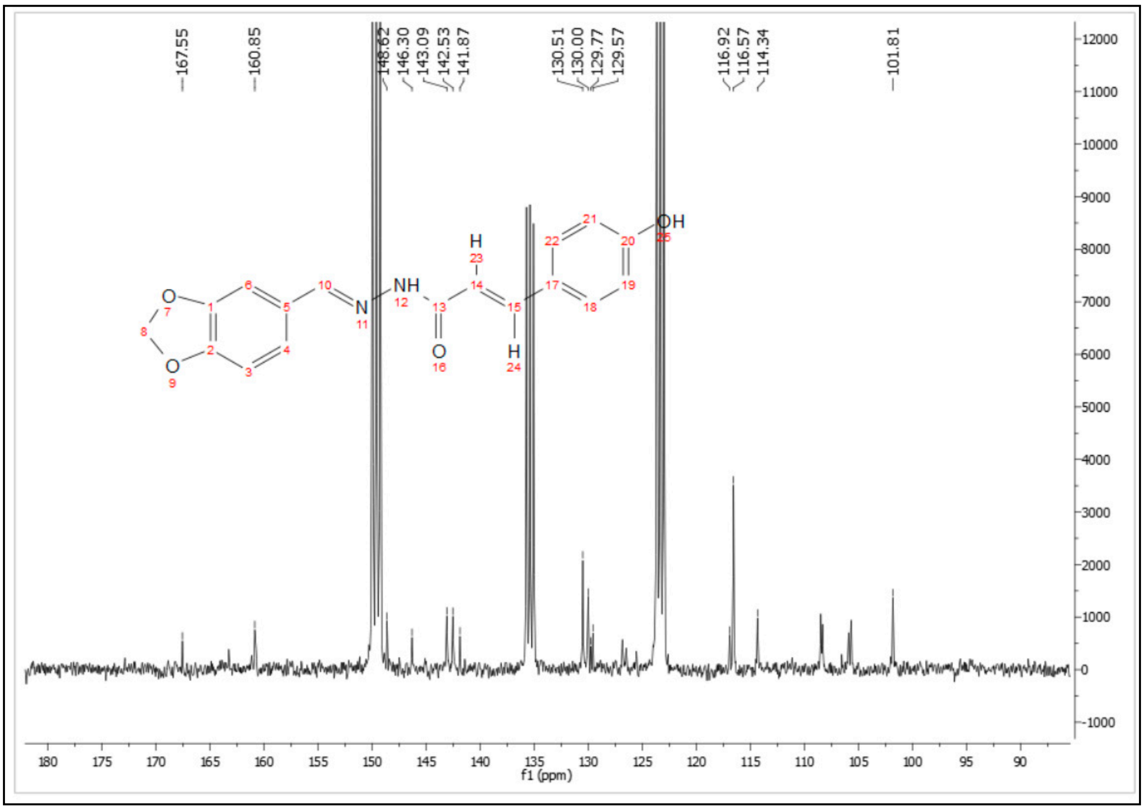

Figure S11 – ^{13}C spectrum (75 MHz, pyridine d_5) of the compound **PQM-280 (3c)**.

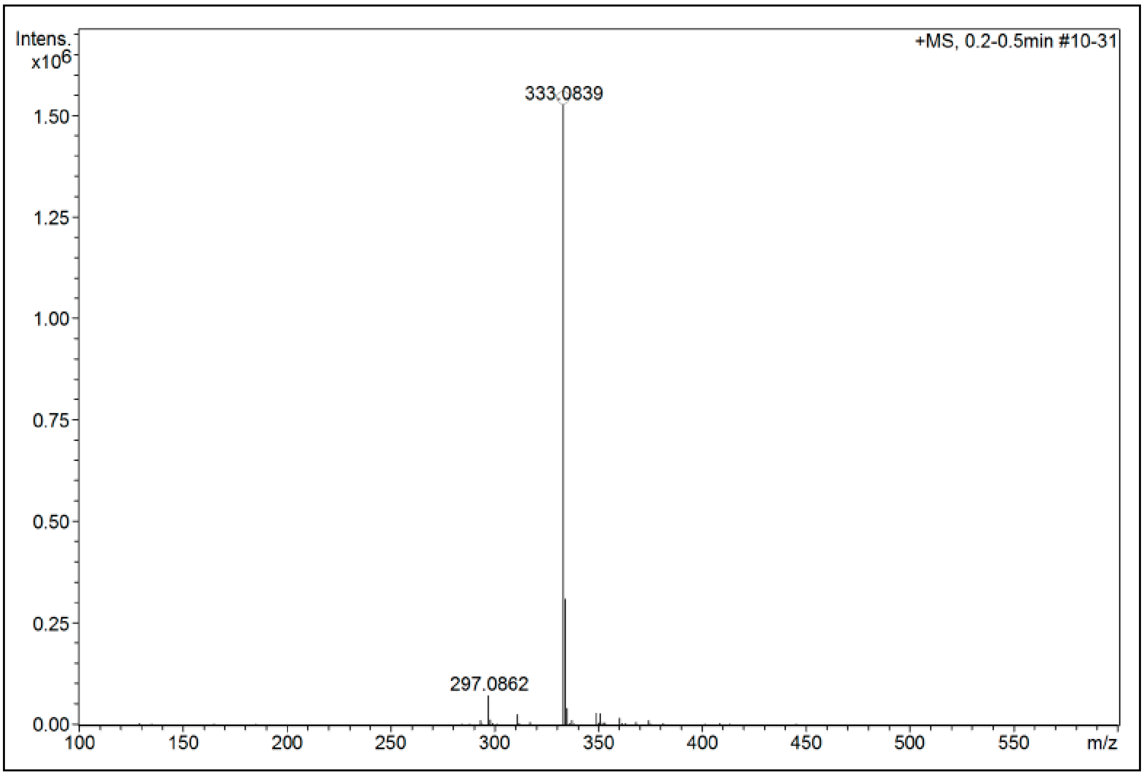

Figure S12 – Mass Spectrum (positive mode) of the compound **PQM-280 (3c)**.

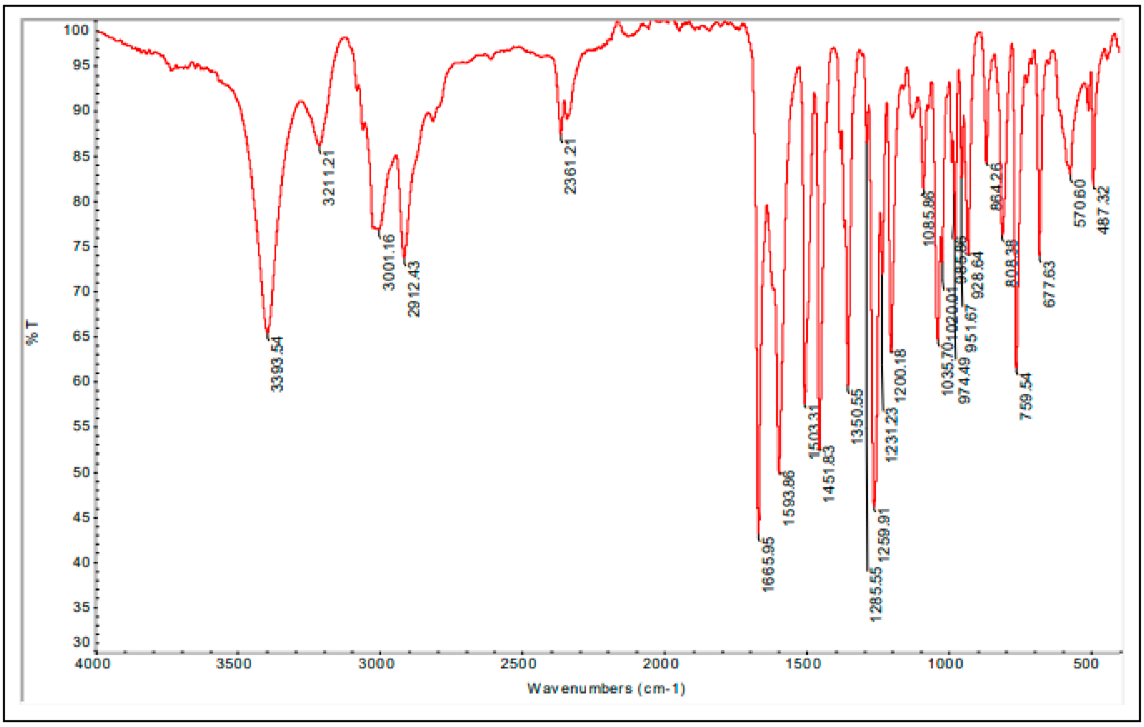

Figure S13 – Infrared absorption spectrum (ATR) of the compound **PQM-281 (3d)**.

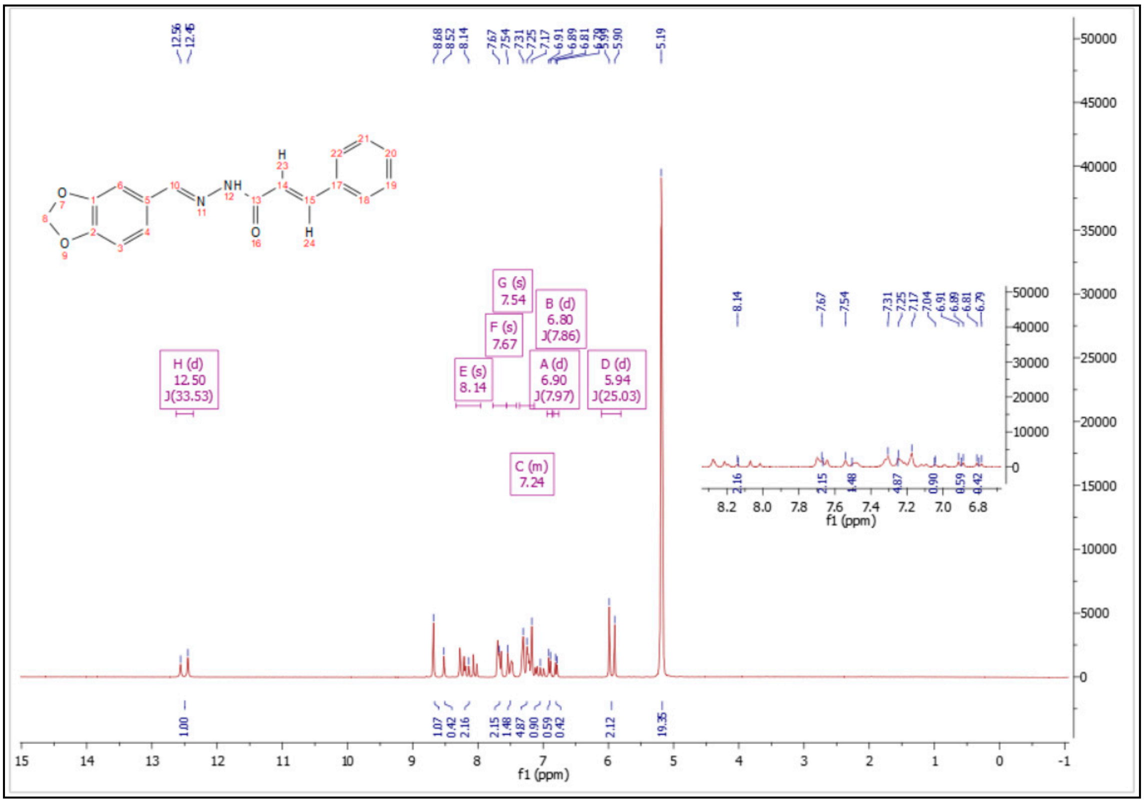

Figure S14 – ^{1}H spectrum (300 MHz, pyridine d_5) of the compound **PQM-281 (3d)**.

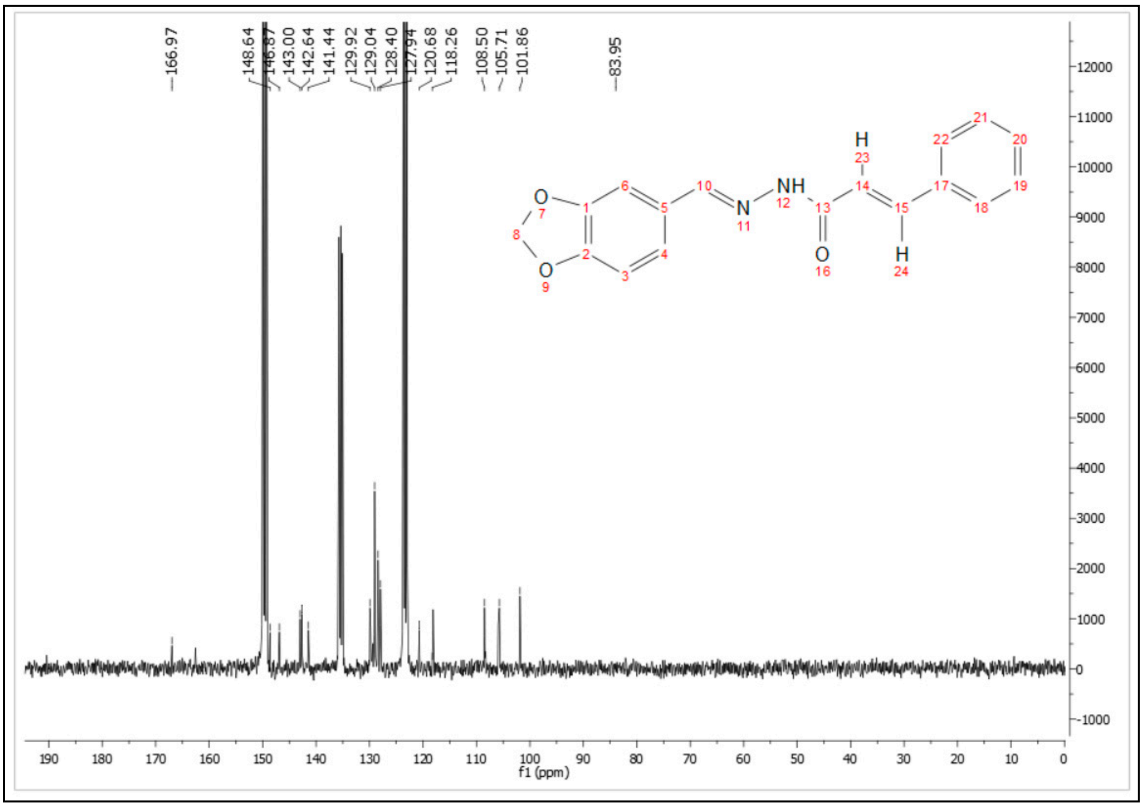

Figure S15 – ^{13}C spectrum (75 MHz, pyridine d_5) of the compound **PQM-281 (3d)**.

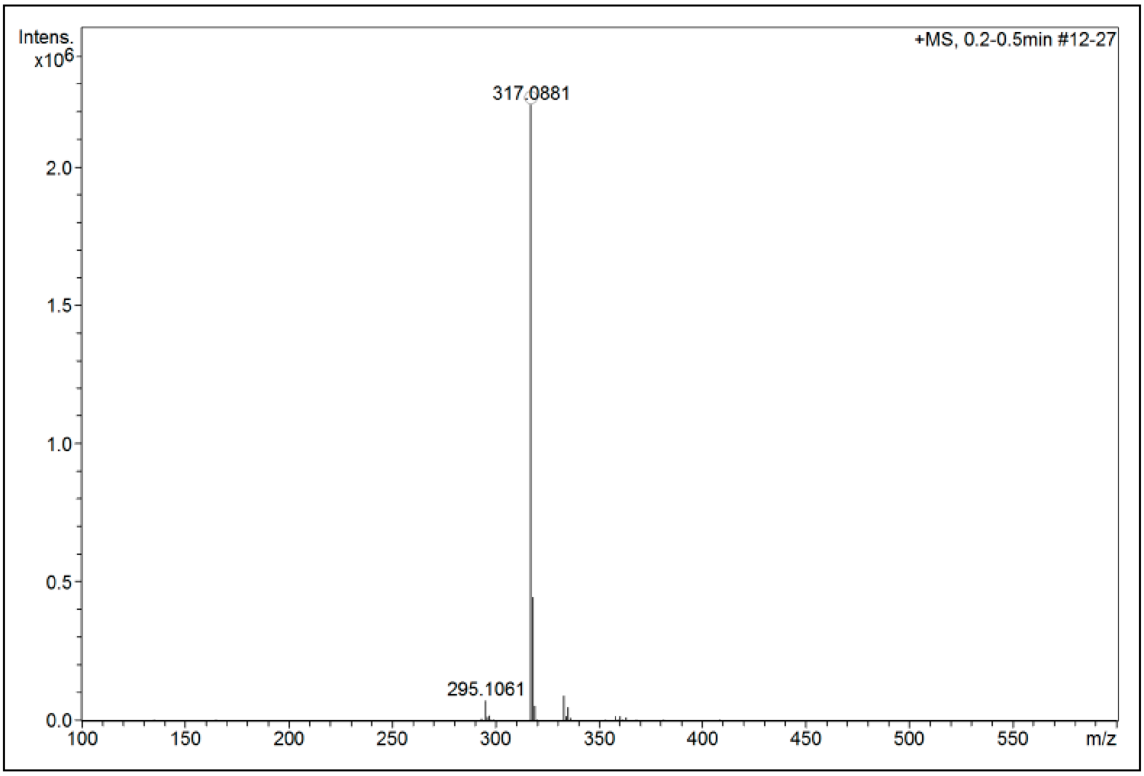

Figure S16 – Mass Spectrum (positive mode) of the compound **PQM-281 (3d)**.

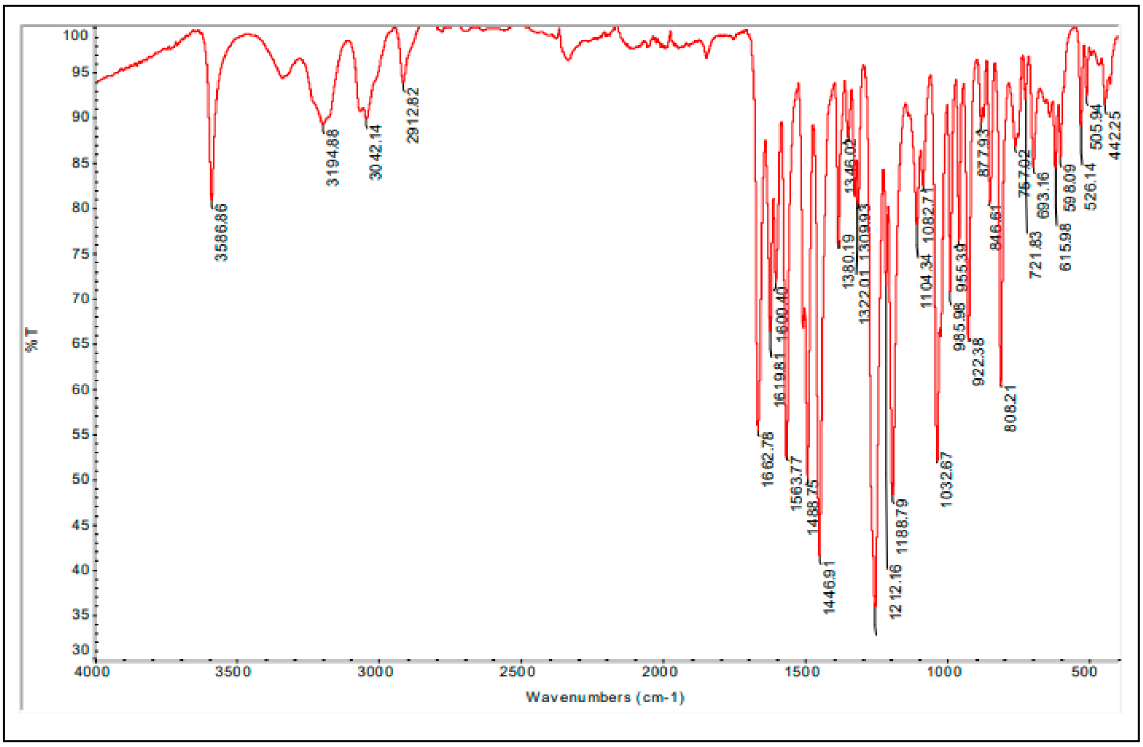

Figure S17 – Infrared absorption spectrum (ATR) of the compound **PQM-284 (3e)**.

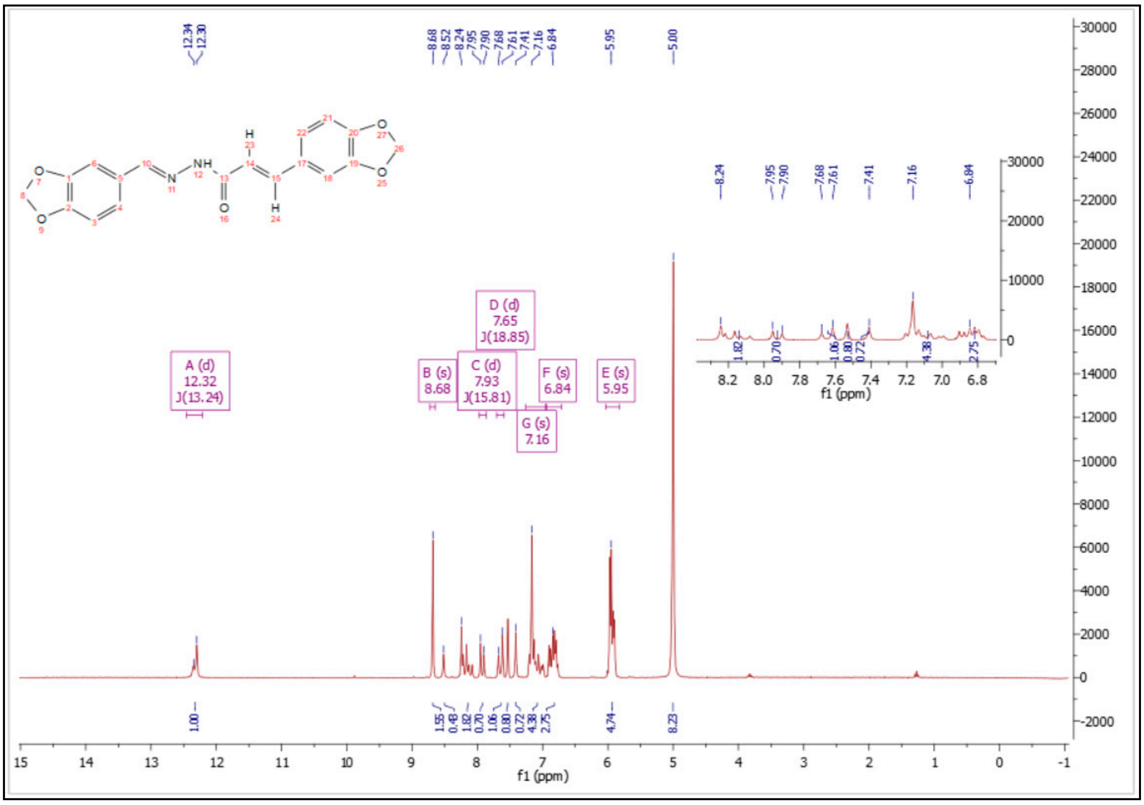

Figure S18 – ^{1}H spectrum (300 MHz, pyridine d_5) of the compound **PQM-284 (3e)**.

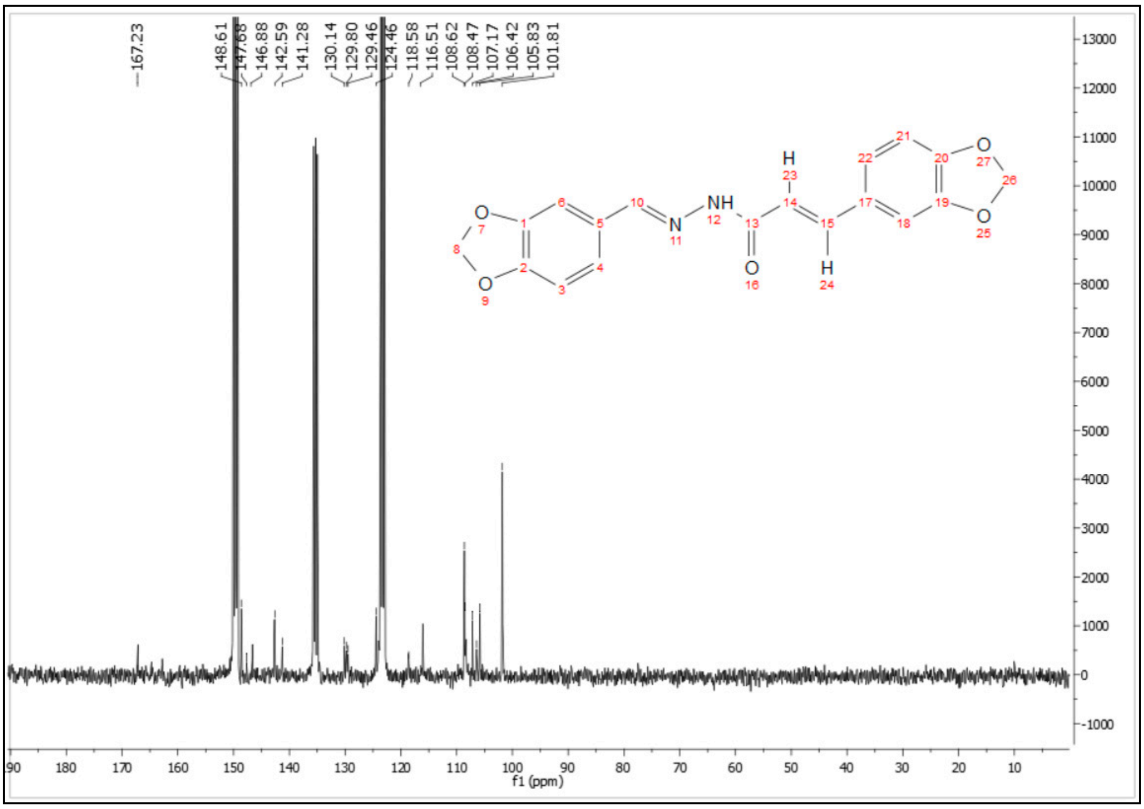

Figure S19 – ^{13}C spectrum (75 MHz, pyridine d_5) of the compound **PQM-284 (3e)**.

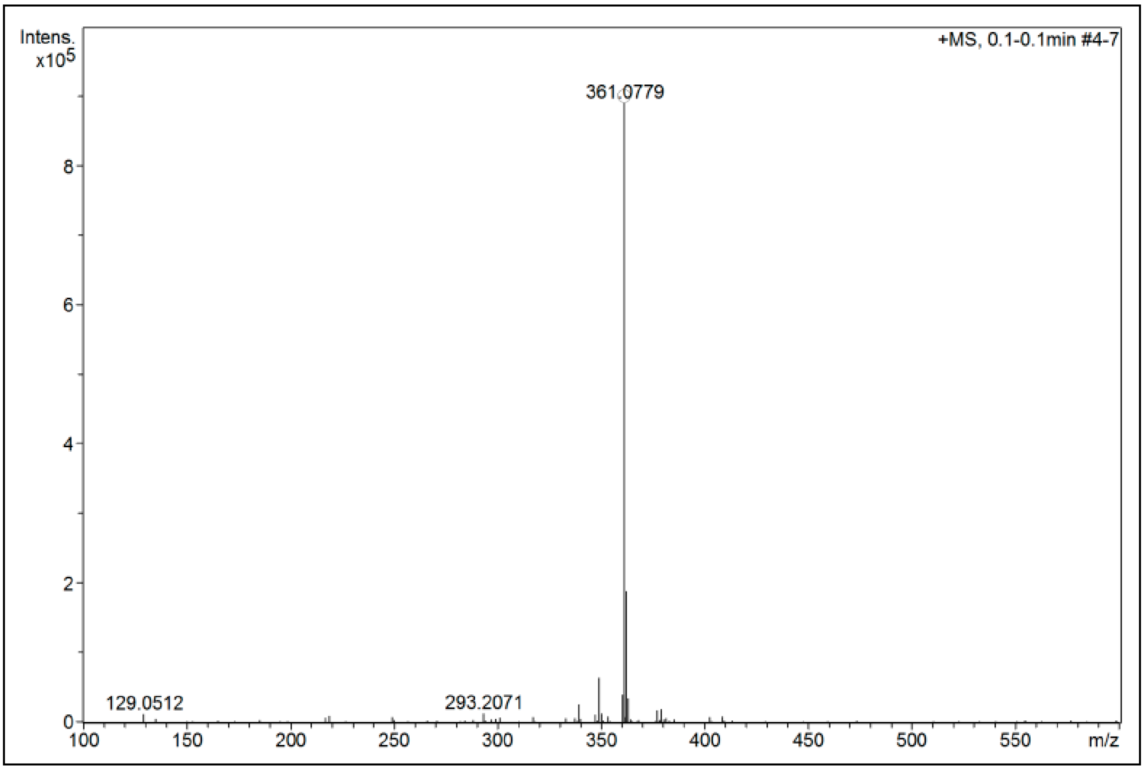

Figure S20 – Mass Spectrum (positive mode) of the compound **PQM-284 (3e)**.

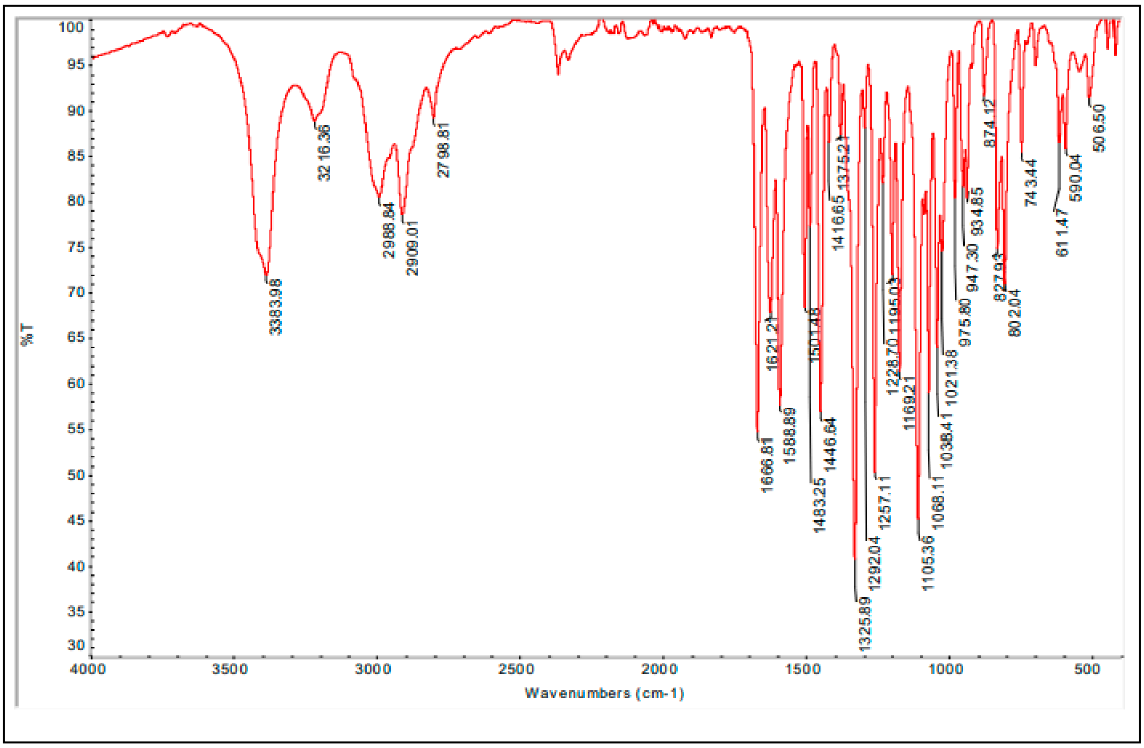

Figure S21 – Infrared absorption spectrum (ATR) of the compound **PQM-285 (3f)**.

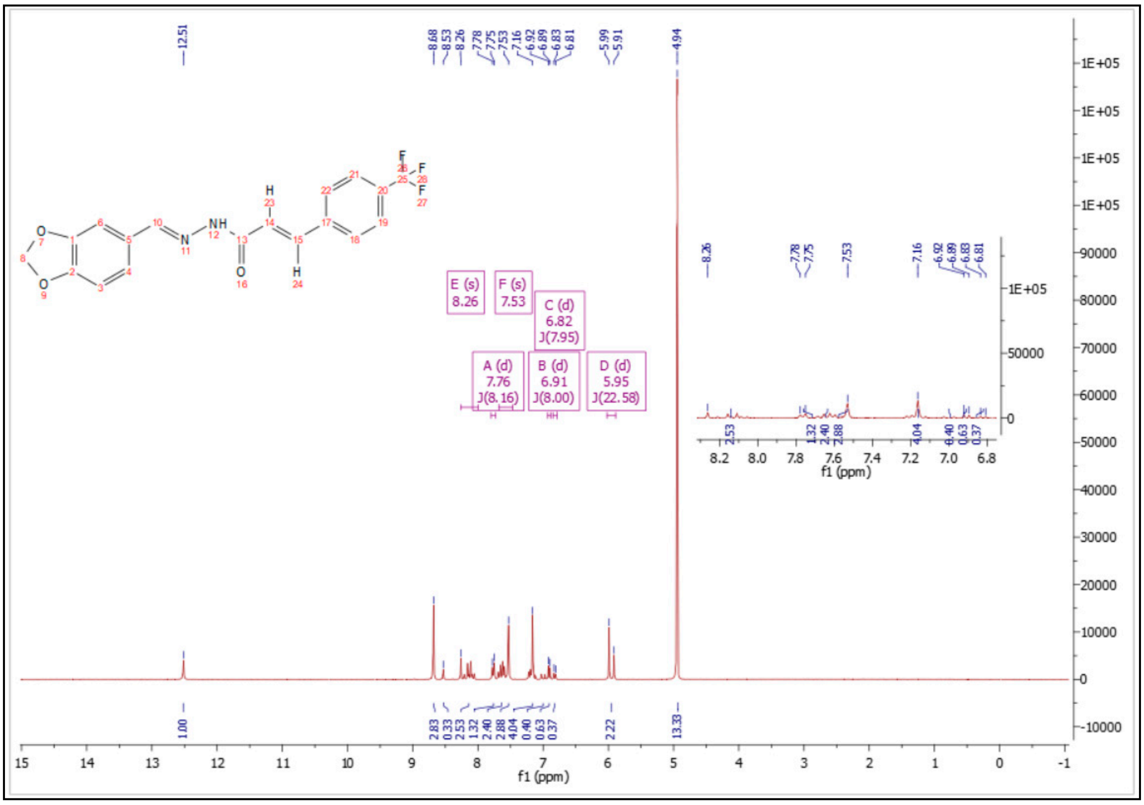

Figure S22 – ^{1}H spectrum (300 MHz, pyridine d_5) of the compound **PQM-285 (3f)**.

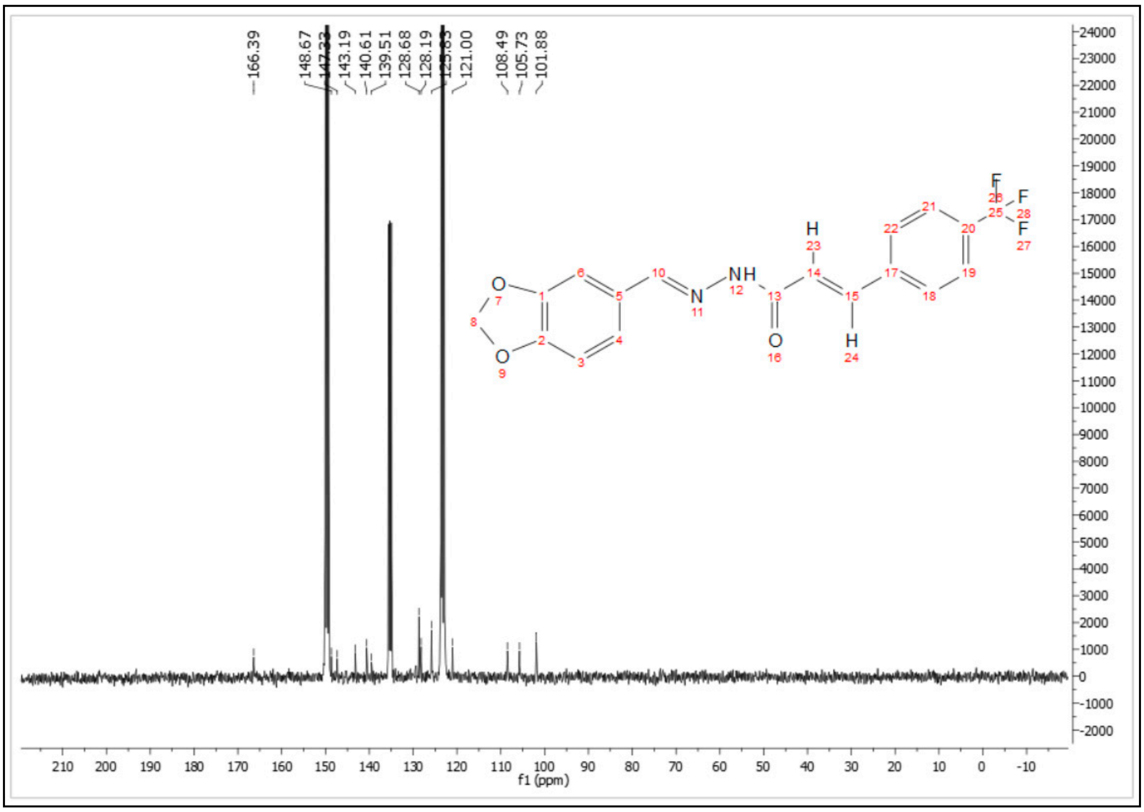

Figure S23 – ^{13}C spectrum (75 MHz, pyridine d_5) of the compound **PQM-285 (3f)**.

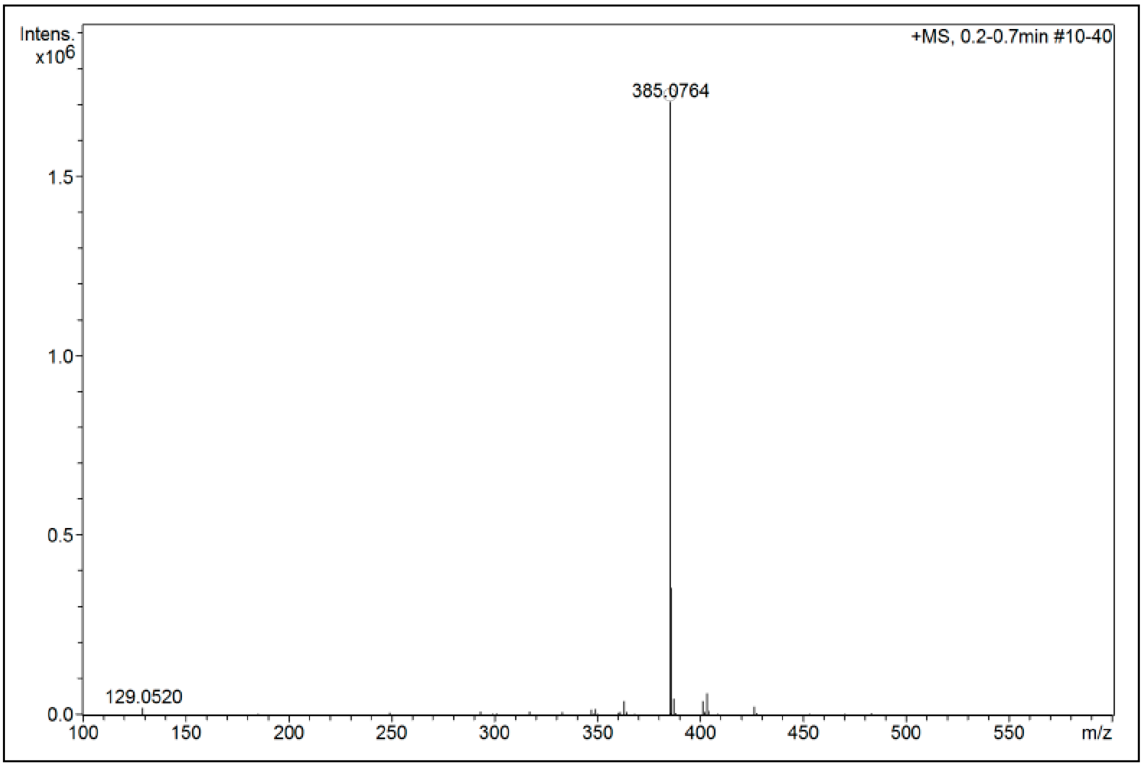

Figure S24 – Mass Spectrum (positive mode) of the compound **PQM-285 (3f)**.

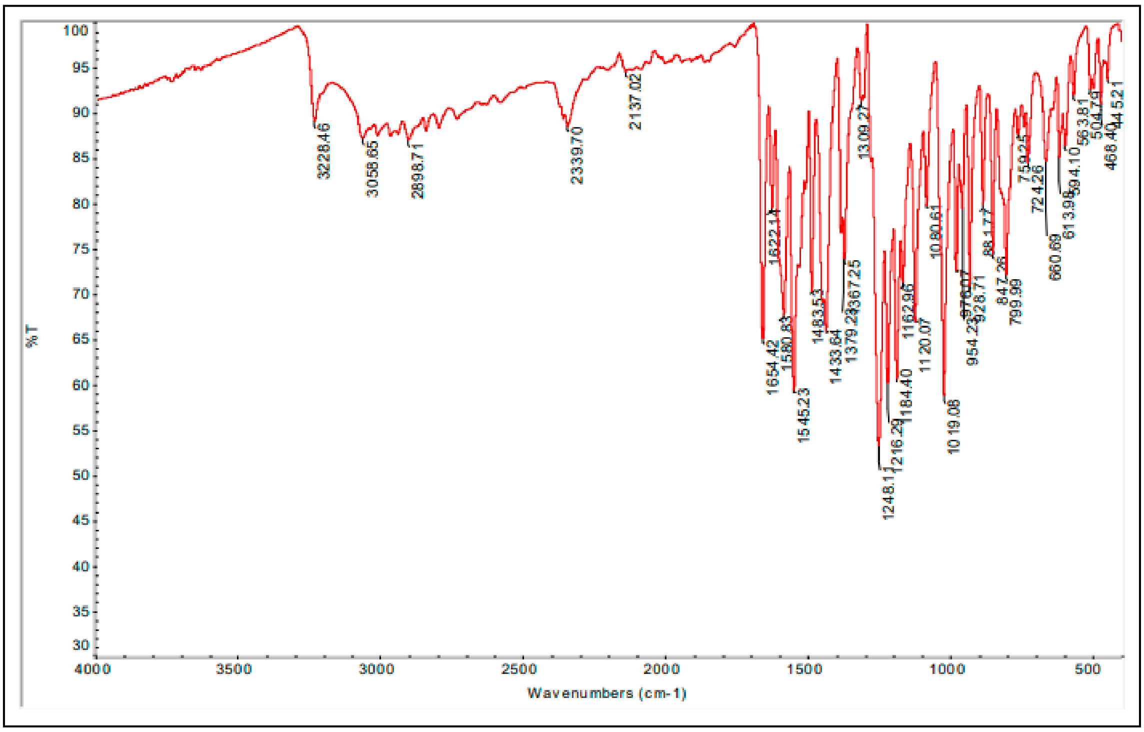

Figure S25 – Infrared absorption spectrum (ATR) of the compound **PQM-286 (3g)**.

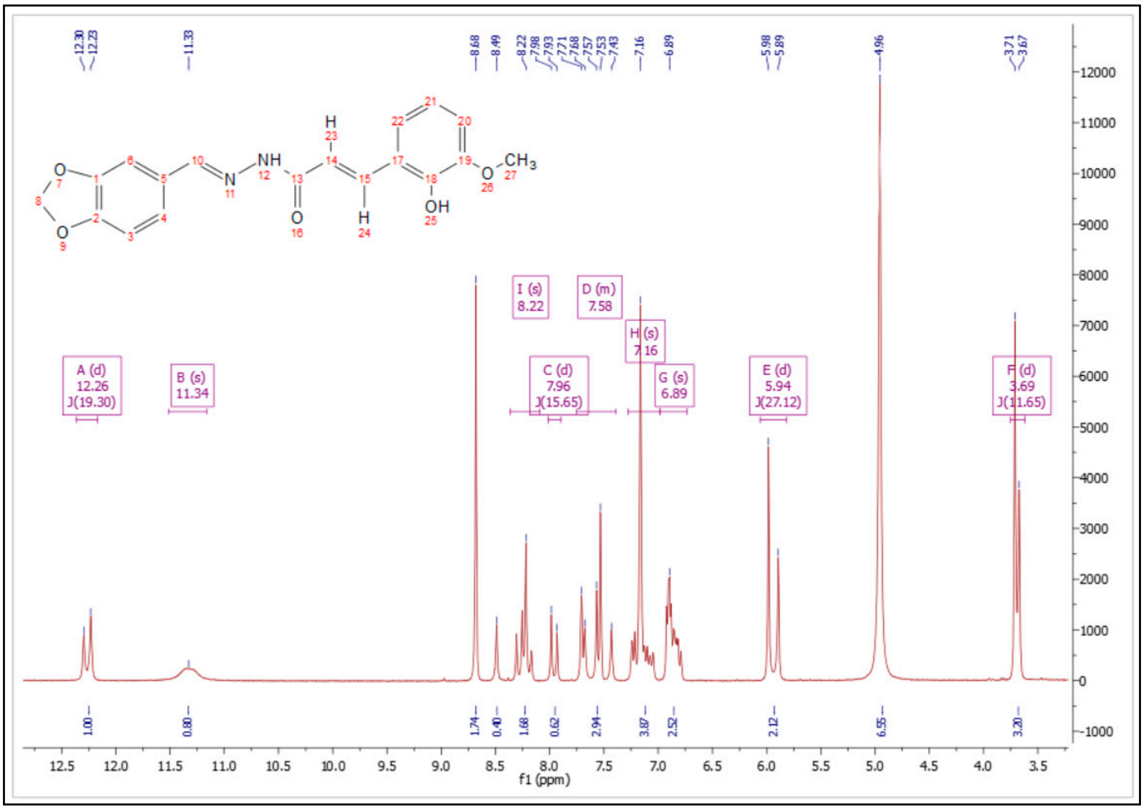

Figure S26 – ^{1}H spectrum (300 MHz, pyridine d_5) of the compound **PQM-286 (3g)**.

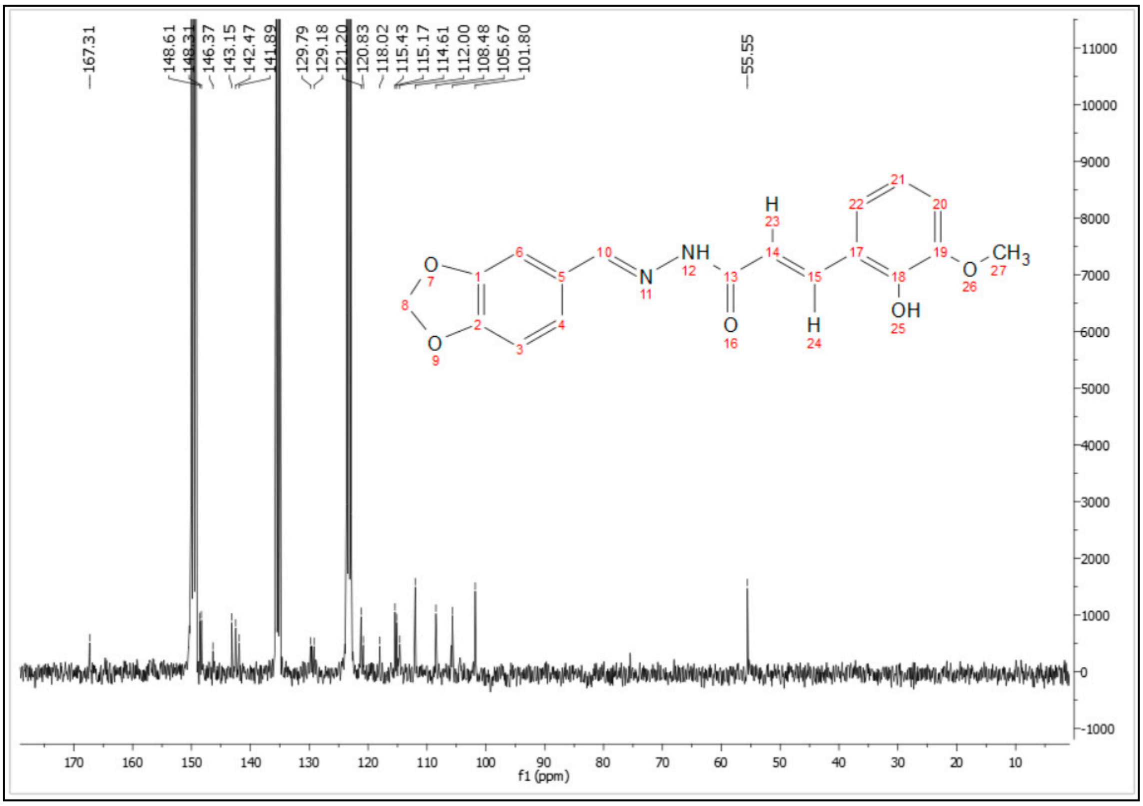

Figure S27 – ^{13}C spectrum (75 MHz, pyridine d_5) of the compound **PQM-286 (3g)**.

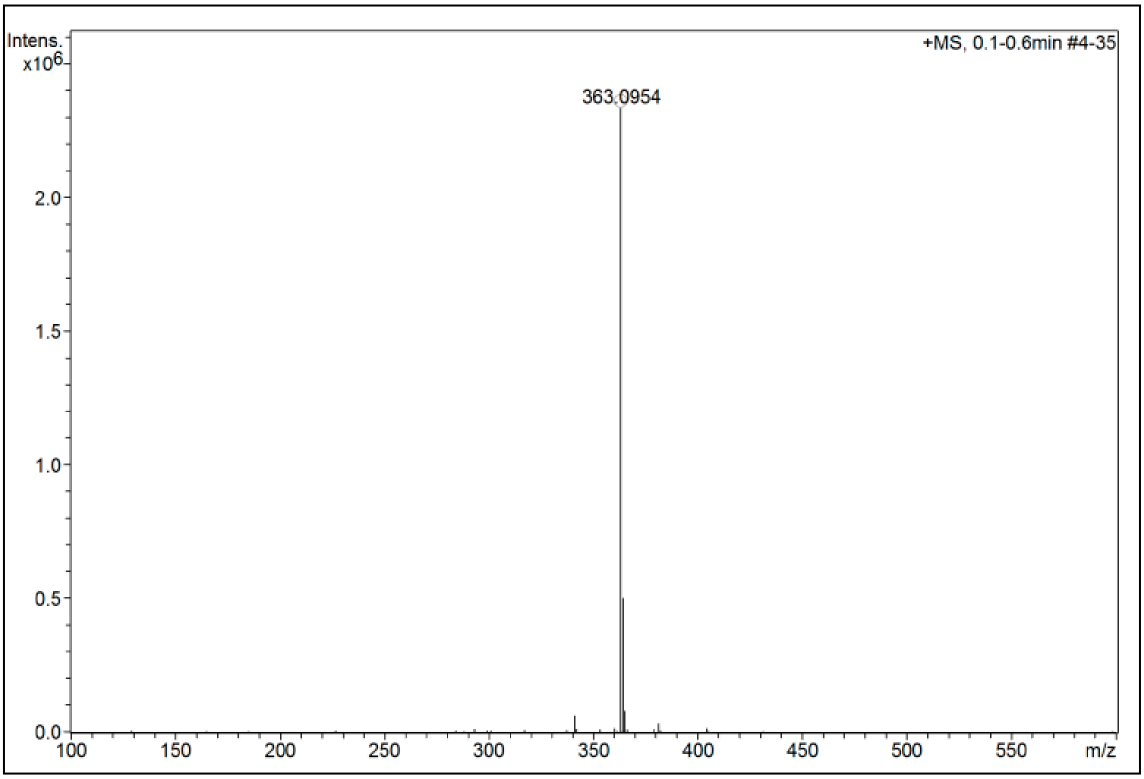

Figure S28 – Mass Spectrum (positive mode) of the compound **PQM-286 (3g)**.

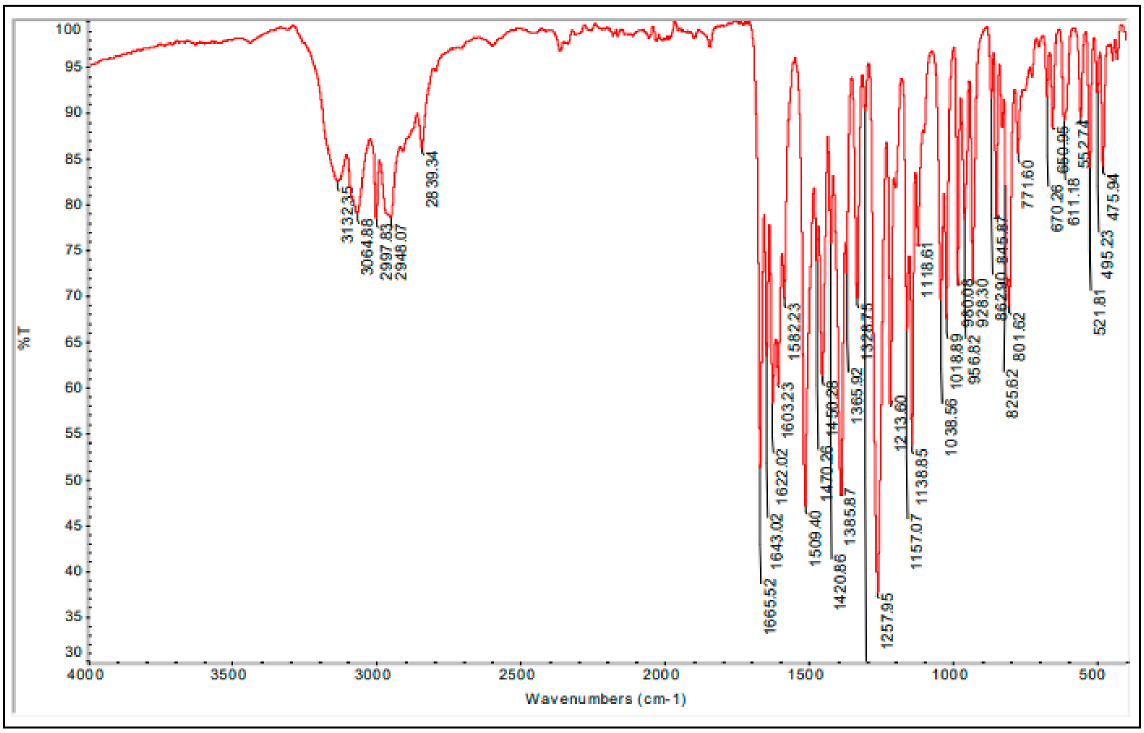

Figure S29 – Infrared absorption spectrum (ATR) of the compound **PQM-287 (3h)**.

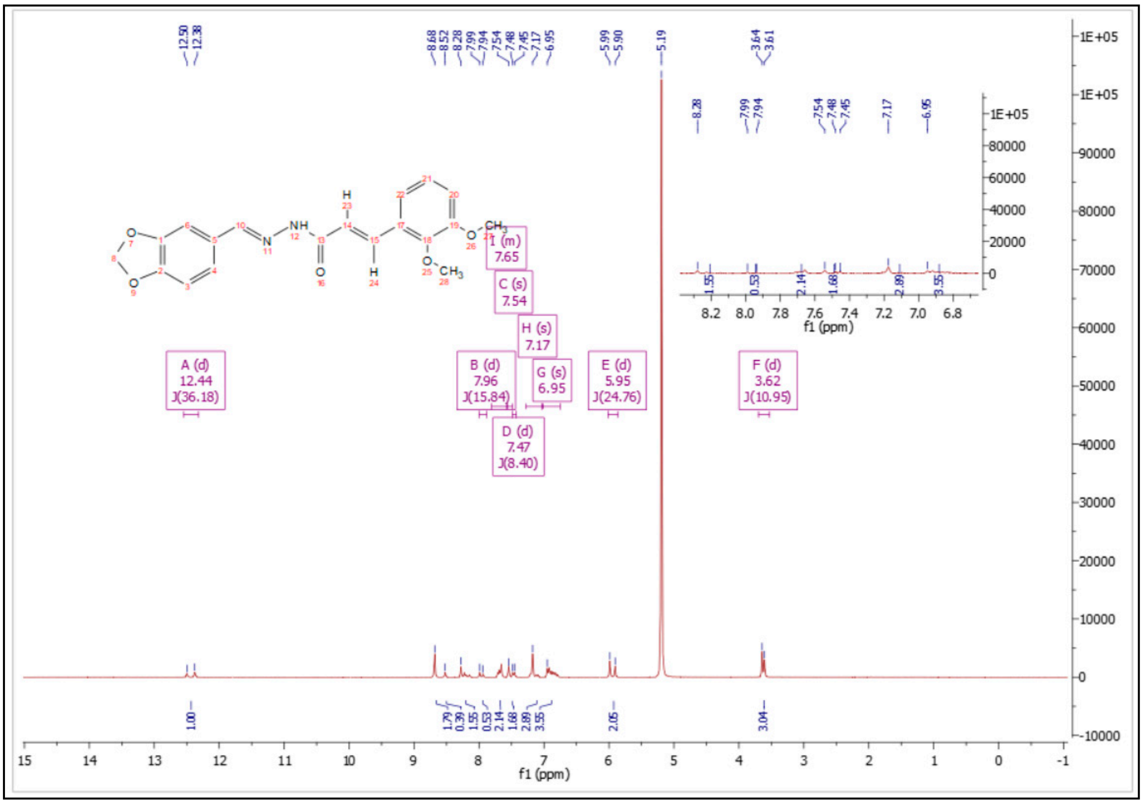

Figure S30 – ^{1}H spectrum (300 MHz, pyridine d_5) of the compound **PQM-287 (3h)**.

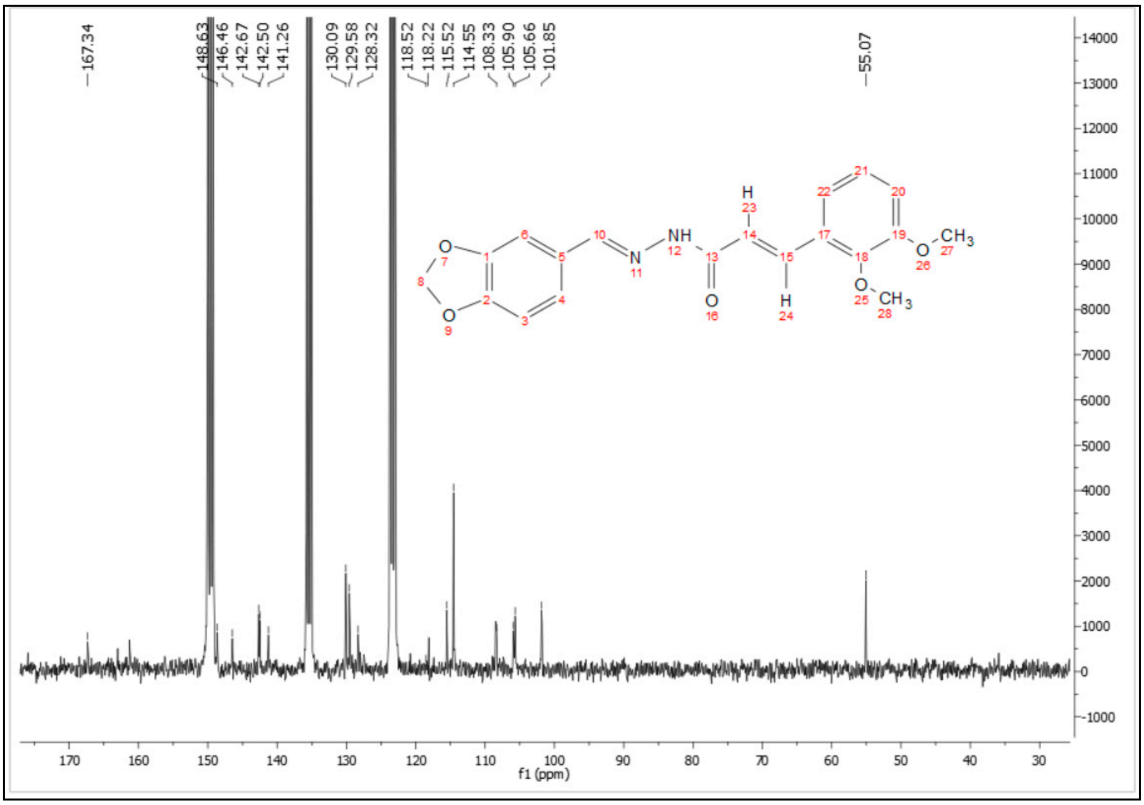

Figure S31 – ^{13}C spectrum (75 MHz, pyridine d_5) of the compound **PQM-287 (3h)**.

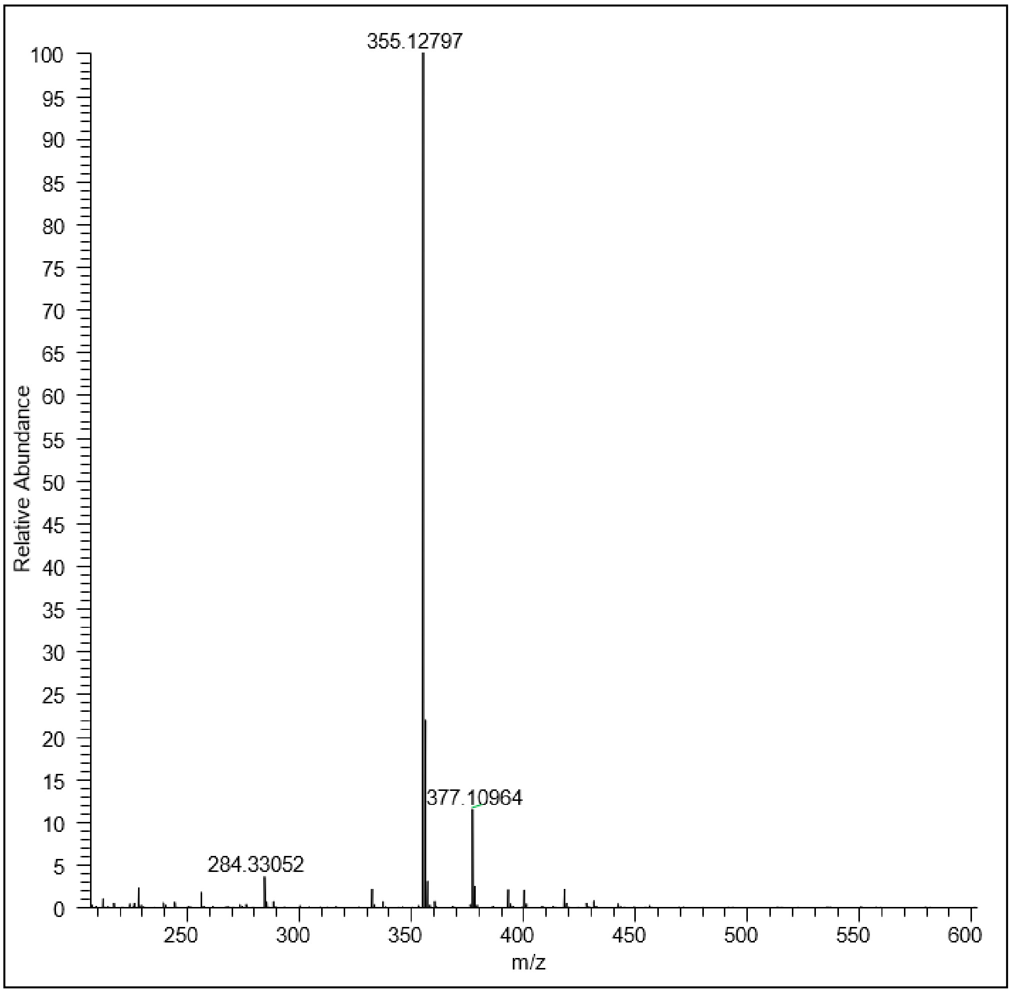

Figure S32 – Mass Spectrum (positive mode) of the compound **PQM-287 (3h)**.

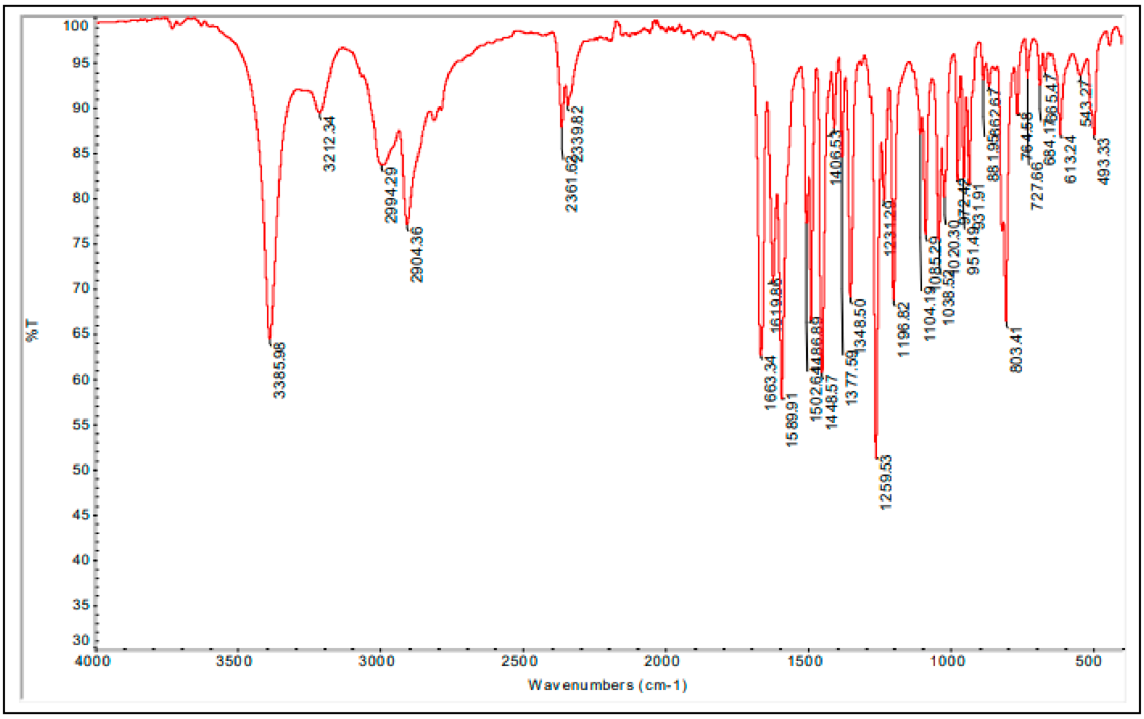

Figure S33 – Infrared absorption spectrum (ATR) of the compound **PQM-288 (3i)**.

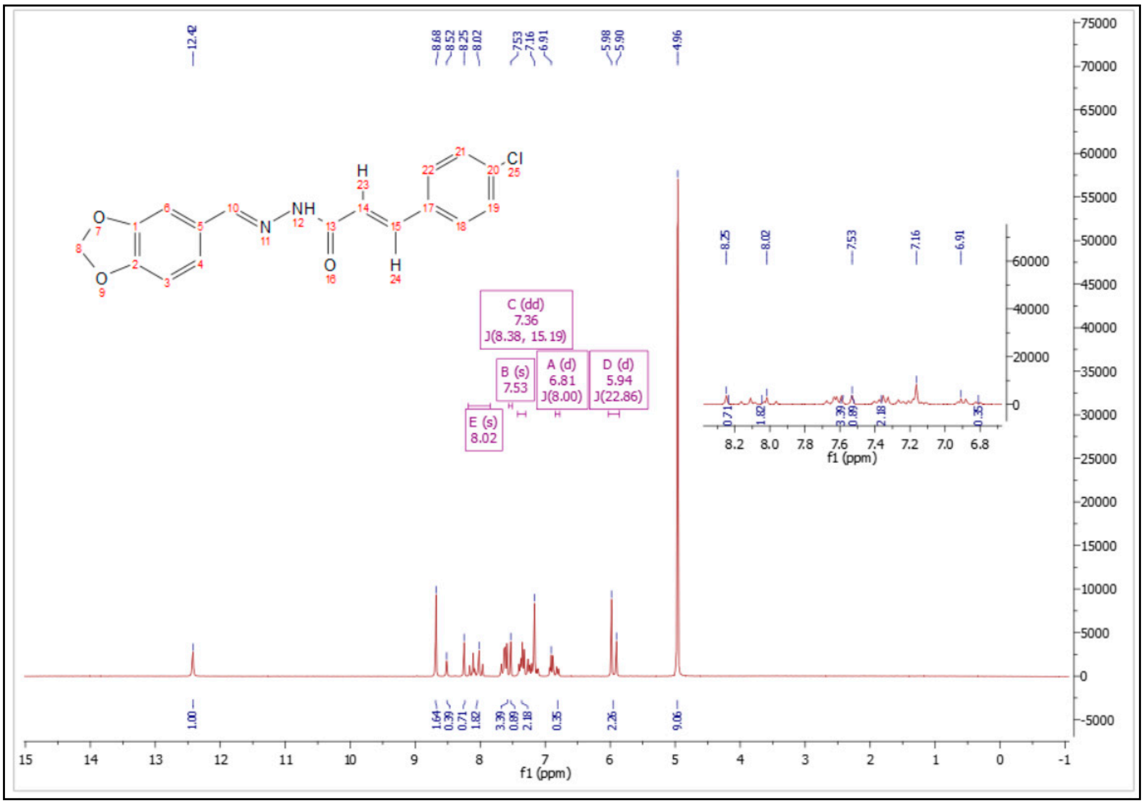

Figure S34 – ^{1}H spectrum (300 MHz, pyridine d_5) of the compound **PQM-288 (3i)**.

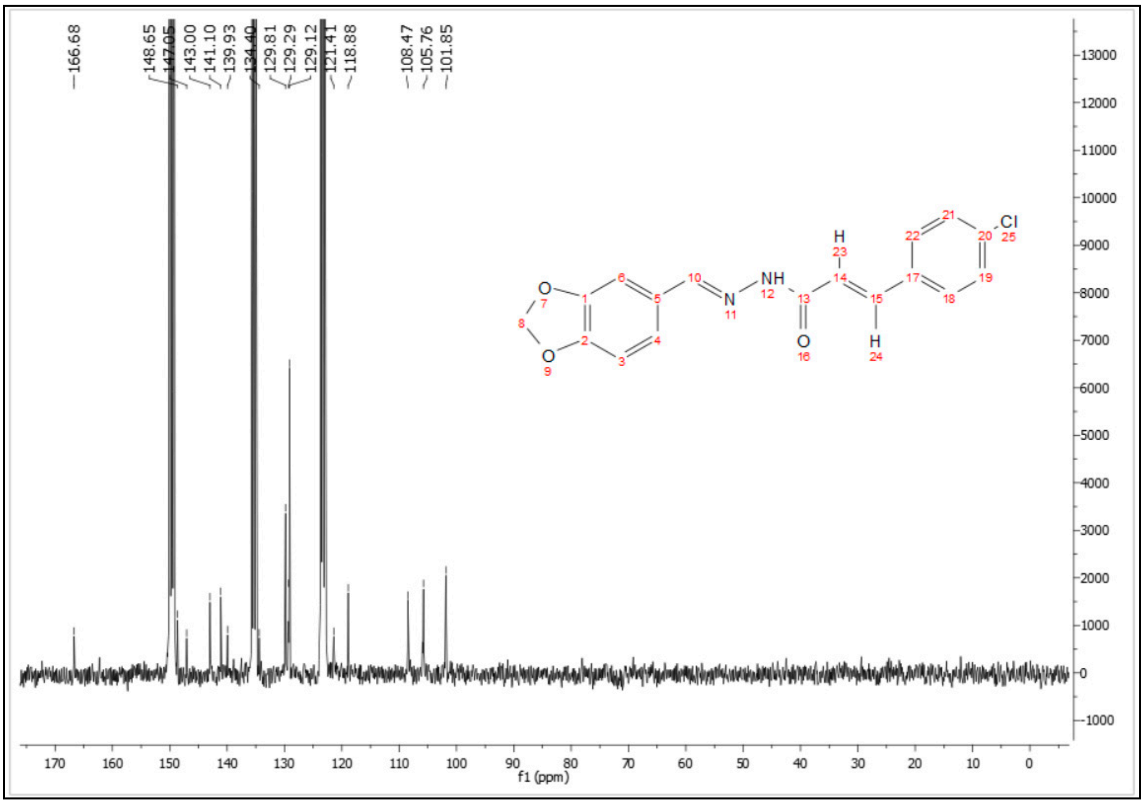

Figure S35 – ^{13}C spectrum (75 MHz, pyridine d_5) of the compound **PQM-288 (3i)**.

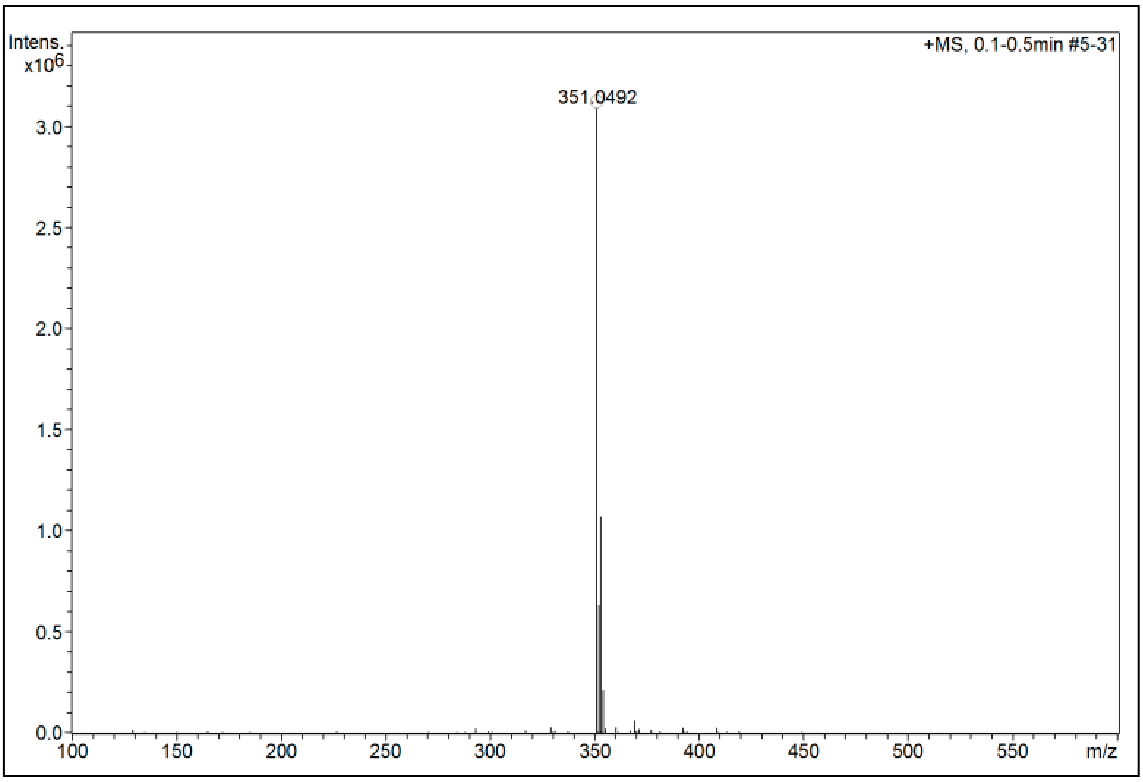

Figure S36 – Mass Spectrum (positive mode) of the compound **PQM-288 (3i)**.
